# Competition and moral behavior: A meta-analysis of forty-five crowd-sourced experimental designs

**DOI:** 10.1073/pnas.2215572120

**Published:** 2023-05-30

**Authors:** Christoph Huber, Anna Dreber, Jürgen Huber, Magnus Johannesson, Michael Kirchler, Utz Weitzel, Miguel Abellán, Xeniya Adayeva, Fehime Ceren Ay, Kai Barron, Zachariah Berry, Werner Bönte, Katharina Brütt, Muhammed Bulutay, Pol Campos-Mercade, Eric Cardella, Maria Almudena Claassen, Gert Cornelissen, Ian G. J. Dawson, Joyce Delnoij, Elif E. Demiral, Eugen Dimant, Johannes Theodor Doerflinger, Malte Dold, Cécile Emery, Lenka Fiala, Susann Fiedler, Eleonora Freddi, Tilman Fries, Agata Gasiorowska, Ulrich Glogowsky, Paul M. Gorny, Jeremy David Gretton, Antonia Grohmann, Sebastian Hafenbrädl, Michel Handgraaf, Yaniv Hanoch, Einav Hart, Max Hennig, Stanton Hudja, Mandy Hütter, Kyle Hyndman, Konstantinos Ioannidis, Ozan Isler, Sabrina Jeworrek, Daniel Jolles, Marie Juanchich, Raghabendra Pratap KC, Menusch Khadjavi, Tamar Kugler, Shuwen Li, Brian Lucas, Vincent Mak, Mario Mechtel, Christoph Merkle, Ethan Andrew Meyers, Johanna Mollerstrom, Alexander Nesterov, Levent Neyse, Petra Nieken, Anne-Marie Nussberger, Helena Palumbo, Kim Peters, Angelo Pirrone, Xiangdong Qin, Rima Maria Rahal, Holger Rau, Johannes Rincke, Piero Ronzani, Yefim Roth, Ali Seyhun Saral, Jan Schmitz, Florian Schneider, Arthur Schram, Simeon Schudy, Maurice E. Schweitzer, Christiane Schwieren, Irene Scopelliti, Miroslav Sirota, Joep Sonnemans, Ivan Soraperra, Lisa Spantig, Ivo Steimanis, Janina Steinmetz, Sigrid Suetens, Andriana Theodoropoulou, Diemo Urbig, Tobias Vorlaufer, Joschka Waibel, Daniel Woods, Ofir Yakobi, Onurcan Yilmaz, Tomasz Zaleskiewicz, Stefan Zeisberger, Felix Holzmeister

**Affiliations:** ^a^Institute for Markets and Strategy, WU Vienna University of Economics and Business, Vienna, Austria; ^b^Department of Economics, Stockholm School of Economics, Stockholm, Sweden; ^c^Department of Economics, University of Innsbruck, Innsbruck, Austria; ^d^Department of Banking and Finance, University of Innsbruck, Innsbruck, Austria; ^e^Department of Finance, School of Business and Economics, Vrije Universiteit Amsterdam, Amsterdam, The Netherlands; ^f^Department of Economics and Business Economics, Nijmegen School of Management, Radboud University, Nijmegen, The Netherlands; ^g^Tinbergen Institute, Amsterdam, The Netherlands; ^h^School of Public Affairs, Leuphana University Lueneburg, Lueneburg, Germany; ^i^HSE University, Saint Petersburg, Russia; ^j^Telenor Research, Telenor Group, Oslo, Norway; ^k^FAIR - The Choice Lab, Norwegian School of Economics, Bergen, Norway; ^l^WZB Berlin Social Science Center, Berlin, Germany; ^m^Department of Organizational Behavior, Industrial and Labor Relations School, Cornell University, Ithaca, NY; ^n^Schumpeter School of Business and Economics, University of Wuppertal, Wuppertal, Germany; ^o^Institute for Development Strategies, Indiana University Bloomington, Bloomington, IN; ^p^Amsterdam School of Economics, University of Amsterdam, Amsterdam, The Netherlands; ^q^Technical University Berlin, Berlin, Germany; ^r^University of Copenhagen, Copenhagen, Denmark; ^s^Rawls College of Business, Texas Tech University, Lubbock, TX; ^t^School of Psychology and Neuroscience, University of Glasgow, Glasgow, Scotland; ^u^Department of Economics and Business, Universitat Pompeu Fabra, Barcelona, Spain; ^v^UPF Barcelona School of Management, Barcelona, Spain; ^w^Centre for Risk Research, University of Southampton, Southampton, United Kingdom; ^x^Section Economics, Wageningen University, Wageningen, The Netherlands; ^y^Department of Accounting, Finance and Economics, Austin Peay State University, Clarksville, TN; ^z^Women and Public Policy Program, Harvard University, Cambridge, MA; ^aa^University of Pennsylvania, Philadelphia, PA; ^bb^Department of Psychology, University of Konstanz, Konstanz, Germany; ^cc^Pomona College, Claremont, CA; ^dd^University of Exeter Business School, Exeter, UK; ^ee^Department of Economics, University of Bergen, Bergen, Norway; ^ff^Institute for Cognition and Behavior, WU Vienna University of Economics and Business, Vienna, Austria; ^gg^Center for Research in Economic Behavior, Institute of Psychology, SWPS University of Social Sciences and Humanities, Wroclaw, Poland; ^hh^Department of Economics, Johannes Kepler University Linz, Linz, Austria; ^ii^Department of Economics and Management, Karlsruhe Institute of Technology, Karlsruhe, Germany; ^jj^Department of Psychology, University of Waterloo, Waterloo, Canada; ^kk^Department of Economics and Business Economics, Aarhus University, Denmark; ^ll^Danish Finance Institute, Denmark; ^mm^Managing People in Organizations Department, IESE Business School, Barcelona, Spain; ^nn^AMS Institute, Amsterdam, The Netherlands; ^oo^School of Business, George Mason University, Fairfax, VA; ^pp^Psychology Department, Eberhard Karls Universität Tübingen, Tübingen, Germany; ^qq^Hankamer School of Business, Baylor University, Waco, TX; ^rr^University of Texas at Dallas, Dallas, TX; ^ss^CREED, University of Amsterdam, Amsterdam, The Netherlands; ^tt^School of Economics, University of Queensland, St Lucia, Australia; ^uu^Faculty of Economics and Management, Otto von Guericke University Magdeburg, Magdeburg, Germany; ^vv^Halle Institute for Economic Research, Halle (Saale), Germany; ^ww^Department of Psychology, University of Essex, Colchester, Uinted Kingdom; ^xx^Rollins College, Winter Park, FL; ^yy^Department of Spatial Economics, School of Business and Economics, Vrije Universiteit Amsterdam, The Netherlands; ^zz^Kiel Institute for the World Economy, Kiel, Germany; ^aaa^Department of Management and Organizations, University of Arizona, Tucson, AZ; ^bbb^Antai College of Economics and Management, Shanghai Jiao Tong University, Shanghai, China; ^ccc^Cambridge Judge Business School, Cambridge, Uinted Kingdom; ^ddd^Interdisciplinary Center for Economic Science, George Mason University, Fairfax, VA; ^eee^Research Institute for Industrial Economics (IFN), Stockholm, Sweden; ^fff^DIW, Berlin, Germany; ^ggg^CESifo, Munich, Germany; ^hhh^Center for Humans and Machines, Max Planck Institute for Human Development, Berlin, Germany; ^iii^Centre for Philosophy of Natural and Social Science, London School of Economics and Political Science, London, Uinted Kingdom; ^jjj^Max Planck Institute for Research on Collective Goods, Bonn, Germany; ^kkk^University of Göttingen, Göttingen, Germany; ^lll^Friedrich-Alexander-Universität Erlangen-Nürnberg, Nürnberg, Germany; ^mmm^International Security and Development Center, Berlin, Germany; ^nnn^University of Haifa, Haifa, Israel; ^ooo^Institute for Advanced Study in Toulous, Toulouse, France; ^ppp^Department of Economics, University of Zurich, Zurich, Switzerland; ^qqq^Department of Economics, LMU Munich, Munich, Germany; ^rrr^The Wharton School, University of Pennsylvania, Philadelphia, PA; ^sss^University of Heidelberg, Heidelberg, Germany; ^ttt^Bayes Business School, City University of London, London, Uinted Kingdom; ^uuu^School of Business and Economics, RWTH Aachen University, Aachen, Germany; ^vvv^Department of Economics, University of Essex, Colchester, Uinted Kingdom; ^www^Working Group Sustainable Use of Natural Resources, University of Marburg, Germany; ^xxx^Department of Economics, Tilburg University, Tilburg, The Netherlands; ^yyy^Institute of Business and Economics, Brandenburg University of Technology Cottbus-Senftenberg, Germany; ^zzz^Institute of Environmental Systems Research and Faculty of Economics and Business Administration, Osnabruck University, Osnabruck, Germany; ^aaaa^Department of Psychology, Kadir Has University, Istanbul, Turkey; ^bbbb^Department of Banking and Finance, University of Zürich, Zürich, Switzerland

**Keywords:** competition, moral behavior, metascience, generalizability, experimental design

## Abstract

Does competition affect moral behavior? This fundamental question has been debated among leading scholars for centuries, and more recently, it has been tested in experimental studies yielding a body of rather inconclusive empirical evidence. A potential source of ambivalent empirical results on the same hypothesis is design heterogeneity—variation in true effect sizes across various reasonable experimental research protocols. To provide further evidence on whether competition affects moral behavior and to examine whether the generalizability of a single experimental study is jeopardized by design heterogeneity, we invited independent research teams to contribute experimental designs to a crowd-sourced project. In a large-scale online data collection, 18,123 experimental participants were randomly allocated to 45 randomly selected experimental designs out of 95 submitted designs. We find a small adverse effect of competition on moral behavior in a meta-analysis of the pooled data. The crowd-sourced design of our study allows for a clean identification and estimation of the variation in effect sizes above and beyond what could be expected due to sampling variance. We find substantial design heterogeneity—estimated to be about 1.6 times as large as the average standard error of effect size estimates of the 45 research designs—indicating that the informativeness and generalizability of results based on a single experimental design are limited. Drawing strong conclusions about the underlying hypotheses in the presence of substantive design heterogeneity requires moving toward much larger data collections on various experimental designs testing the same hypothesis.

## Significance

Using experiments involves leeway in choosing one out of many possible experimental designs. This choice constitutes a source of uncertainty in estimating the underlying effect size which is not incorporated into common research practices. This study presents the results of a crowd-sourced project in which 45 independent teams implemented research designs to address the same research question: Does competition affect moral behavior? We find a small adverse effect of competition on moral behavior in a meta-analysis involving 18,123 experimental participants. Importantly, however, the variation in effect size estimates across the 45 designs is substantially larger than the variation expected due to sampling errors. This “design heterogeneity” highlights that the generalizability and informativeness of individual experimental designs are limited.

Does competition erode, promote, or not affect moral behavior? This fundamental question has been debated since the early history of modern economics and in the social sciences in general. Adam Smith argued that markets, which are inherently competitive, would have a civilizing effect on participants’ behavior (1, 2). In line with this, some modern-day scholars argue that markets may reduce conflict and violence (3), enhance morality, and induce trust and prosocial behavior (4–6). In contrast, Karl Marx (7) and Thorstein Veblen (8) expected market activity to be inherently alienating and cause ills such as dishonesty, bringing out the worst in human beings. Also today, some economists argue that competitive pressure may create strong incentives for unethical practices (like child labor, tax evasion, or corruption) and undermine moral values per se by crowding out social norms (9, 10).

Recently, this debate has been taken to the laboratory, where controlled experiments promise causal inference. In a seminal study, more participants were willing to give up money for preventing the death of a mouse when making decisions individually than when competing in markets (11). However, follow-up studies, relying on alternative designs, question the robustness and interpretation of this finding and provide inconclusive evidence on the interplay of competitive markets and moral behavior (12–16). In addition, studying the impact of competition on behavior, particularly on prosocial and moral aspects, has gained growing attention in a variety of disciplines such as management, psychology, organizational studies, and sociology (17, 18).

The previous literature points to two main challenges in testing if competition affects moral behavior: how to experimentally implement competition and how to measure whether observed behavior is moral? As there are multiple valid approaches to address both issues, we implemented a crowd-sourced research program (19). We invited research teams (RTs) to independently design and implement an online experiment with one competition and one control condition. We left it to the RTs how to operationalize competition and how to elicit and assess moral behavior, but they were required to use an “experimental economics protocol” with monetary incentives and no deception (20). After screening the initial applications based on our preregistered inclusion criteria, 102 RTs were invited to submit a research design, 95 RTs submitted a research design, of which 50 RTs were randomly selected to participate in the study, and of which, 45 RTs delivered the experimental software and were thus included in the data collection. Participants in the experiments were randomly assigned to one of the 45 × 2 experimental treatments, and 18,123 completed observations (about 400 per design) were collected via Prolific in January 2022. The average age of the Prolific participants who completed the experiment was 32.5 y (*sd* = 12.3), and 55.5% were female. Of the participants, 32.5% were from the United Kingdom, 15.1% from the United States, and 11.7% from South Africa. The average Prolific approval rate of the participants was 99.5 (*sd* = 1.0), and English is the first language for 48.1% of the sample. Of the participants, 34.4% are fully employed, and 39.2% are students. We do not find evidence of systematic differences in individual characteristics of the participants who completed the experiment across the 45 research designs (see the *Materials and Methods* section and *SI Appendix*, Fig. S1 for details).

The research design underlying our study allows us to accomplish two important goals. First, we can estimate a meta-analytic effect size across 45 experimental designs—proposed by 45 independent RTs around the globe—that is free from p-hacking, publication bias, and “hypothesizing after results are known” (HARKing) (21). Relying on common research practices, accumulating 45 studies on the same research question would likely take several years, and the resulting sample of studies would potentially be biased due to questionable research practices and the file drawer problem (21). Second, the methodology of our study allows us to isolate the effect of design heterogeneity—the variation in the true effect size across experimental designs—and to estimate its magnitude and to assess its practical relevance. Design heterogeneity is a fundamental methodological concept with immediate implications for the generalizability of experimental results, but it has attracted limited attention in the existing literature (22). Particularly, design heterogeneity constitutes an additional layer of uncertainty in the estimation of the effect size that is not considered by statistical inference practices, and that is inversely related to the generalizability of experimental findings, i.e., high design heterogeneity implies low generalizability.

## Results

Our hypotheses and tests follow a detailed preanalysis plan (PAP) registered prior to starting the data collection (osf.io/r6anc). During the review process, the editor and two anonymous reviewers suggested additional exploratory analyses and robustness tests; we are grateful for the suggested amendments. All paragraph headings clearly indicate whether or not the corresponding analyses were preregistered. As preregistered, we use the threshold of *P* < 0.005 for “statistically significant evidence” and *P* < 0.05 for “suggestive evidence” (23).

### Meta-Analytic Effect Size (Preregistered).

Our first primary hypothesis is that competition affects moral behavior. This hypothesis is not directional, as different scholars have argued for both a negative and a positive effect of competition on moral behavior, and the existing empirical literature is also inconclusive about whether the eventual effect of competition is positive or negative. We test this hypothesis using the pooled evidence of the 45 crowd-sourced research designs and analyze the data using two analytic approaches (A and B). In analytic approach A, the meta-analytic effect size and its standard error are based on the analyses preregistered by the RTs. In analytic approach B, we standardize the analyses across RTs to avoid any variation across designs due to different analytical decisions.

**Fig. 1.**
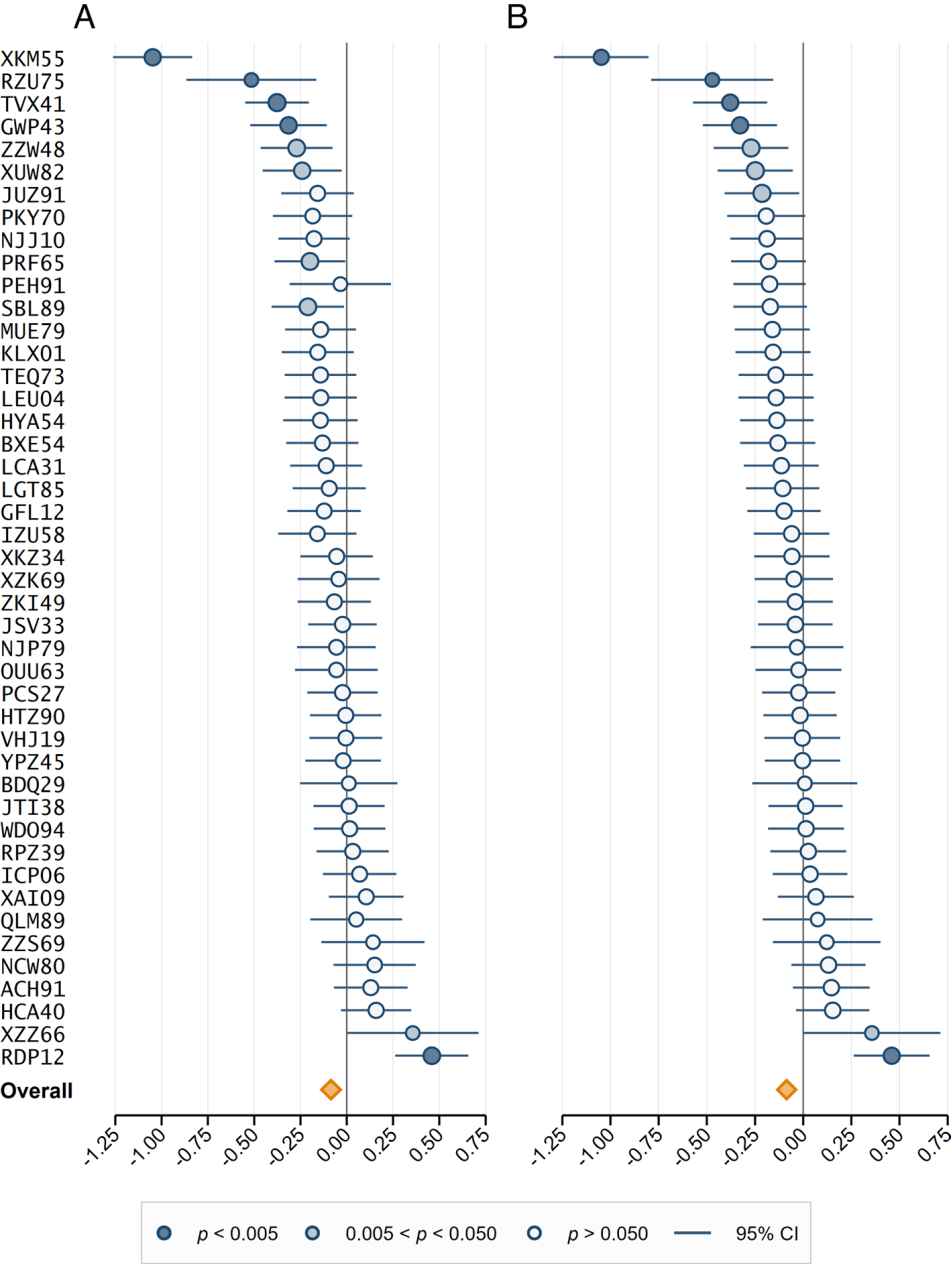
Forest plot of meta-analytic results. (*A*) Plotted are the point estimates and the 95% CIs of the effect sizes in the 45 experimental designs and a random-effects meta-analysis for analytic approach A (in Cohen’s *d* units). There is statistically significant evidence (*P* < 0.005) of a negative effect of competition on moral behavior in four of the individual designs and suggestive evidence (*P* < 0.05) in four additional designs, and there is statistically significant evidence (*P* < 0.005) of a positive effect of competition on moral behavior in one of the individual designs and suggestive evidence (*P* < 0.05) in one additional design. There is suggestive evidence of an adverse effect of competition on moral behavior in the meta-analysis (*d* = −0.085, 95% CI [−0.147, −0.022], *P* = 0.008). (*B*) Plotted are the point estimates and the 95% CIs of the effect sizes in the 45 experimental designs and a random-effects meta-analysis for analytic approach B (in Cohen’s *d* units). There is statistically significant evidence (*P* < 0.005) of a negative effect of competition on moral behavior in four of the individual designs and suggestive evidence (*P* < 0.05) in three additional designs, and there is statistically significant evidence (*P* < 0.005) of a positive effect of competition on moral behavior in one of the individual designs and suggestive evidence (*P* < 0.05) in one additional design. There is statistically significant evidence of an adverse effect of competition on moral behavior in the meta-analysis (*d* = −0.086, 95% CI [−0.144, −0.027], *P* = 0.004).

Fig. 1 shows the effect sizes and the 95% confidence interval of the 45 experimental designs (see *SI Appendix*, Tables S1 and S2 for details) and the meta-analytic effect size (24) based on a random-effects model. The meta-analytic effect size in Cohen’s *d* units is −0.085 (95% CI [−0.147, −0.022], *P* = 0.008) for analytic approach A and −0.086 (95% CI [−0.144, −0.027], *P* = 0.004) for analytic approach B. We thus find suggestive evidence for a negative effect of competition on moral behavior for analytic approach A and statistically significant evidence for analytic approach B. Yet, for both analytical approaches, the magnitude of the effect turns out to be small.

### Design Heterogeneity (Preregistered).

Our second primary hypothesis is that effect size estimates vary across research designs over and above the variation expected from pure sampling variation (i.e., the within-study variance). We refer to this kind of heterogeneity in effect sizes as “design heterogeneity,” and it is measured as the between-study variance in true effect sizes across the experimental designs in the random-effects meta-analysis. Previous work has documented low to moderate heterogeneity in effect sizes across populations (25–27) and substantial heterogeneity in effect sizes across analytical decisions (28–31); however, for design heterogeneity, systematic evidence is scarce (22). We eliminate population heterogeneity by randomly allocating participants to different designs, and we preempt analytical heterogeneity by standardizing the analyses across designs in analytic approach B. Thus, by design, any variation not attributable to sampling variation (i.e., a study’s SE) is due to design heterogeneity.

Based on Cochran’s *Q* tests, we find statistically significant heterogeneity for both analytic approach A (*Q*(44) = 181.1, *P* < 0.001) and analytic approach B (*Q*(44) = 161.5, *P* < 0.001). The heterogeneity measured in a random-effects meta-analysis reflects variation in true effect sizes across studies (between-study variance), which cannot be explained by random error (within-study variance) (32). As analytic approach B removes other sources of heterogeneity, our results provide strong evidence of design heterogeneity, i.e., that the true effect sizes vary across the 45 experimental designs above and beyond the variation that would be expected due to chance alone. According to the estimated *I^2^* for analytic approach B, 72.8% (95% CI [64.8, 85.8]) of the variation in results across research designs is attributable to design heterogeneity; the corresponding result for analytic approach A is 75.7% (95% CI [66.2, 86.0]). A limitation of the *I^2^* measure is that it depends on how precisely the effect size is estimated for each individual research design; the larger the sample size per research design, the larger the share of the variation explained by heterogeneity. The estimated *τ*, which is our preferred measure for quantifying heterogeneity, implies that the standard deviation of the true effect size across research designs is 0.185 (95% CI [0.147, 0.259]) and 0.169 (95% CI [0.140, 0.254]) for analytic approaches A and B, respectively. This is 69% larger than the average sampling standard error of the individual designs for analytic approach A and 57% larger than the average sampling standard error for analytic approach B.

### Moderating Effects of Research Quality (Preregistered).

**Fig. 2.**
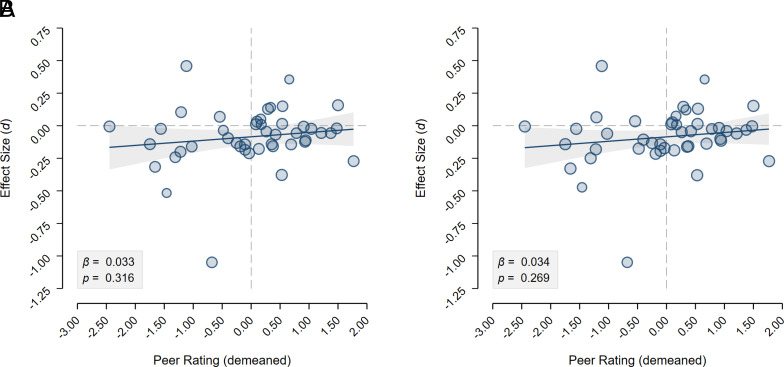
Relationship between effect sizes and experimental design quality. (*A*) Plotted are the 45 estimated effect sizes in analytic approach A over the average (demeaned) quality ratings of the experimental designs. The linear relationship between the two variables estimated using a meta-regression is also plotted together with its 95% CI, revealing no systematic relationship (*b* = 0.033, *se* = 0.033, *P* = 0.316; *R^2^* = 0.000). (*B*) Plotted are the 45 estimated effect sizes in analytic approach B over the average (demeaned) quality ratings of the experimental designs. The linear relationship between the two variables estimated using a meta-regression is also plotted together with its 95% CI, revealing no systematic relationship (*b* = 0.034, *se* = 0.031, *P* = 0.269; *R^2^* = 0.000).

We also test two secondary hypotheses related to the quality of the research designs. Before the data collection, each RT anonymously assessed the quality of ten other experimental designs on a 0 to 10 scale. The average rated quality is 6.0 (*sd* = 1.0, *n* = 45). Our first secondary hypothesis is that the effect sizes vary systematically with the average quality ratings. In Fig. 2, we plot the estimated effect sizes of the 45 designs against the average (demeaned) quality assessments. Fig. 2 reveals no evident pattern between effect size estimates and quality ratings, which is confirmed by formally testing this hypothesis in a meta-regression. In secondary hypothesis 2, we test whether there is heterogeneity in the estimated effect sizes across research designs after controlling for the rated quality. As the quality ratings do virtually not explain any of the variation in effect sizes, heterogeneity remains statistically significant for both analytic approaches (see *SI Appendix*, section S3 for details).

### Exploratory and Robustness Analyses (Preregistered).

We also estimate the meta-analytic effect and the heterogeneity measures for the 50% of experimental designs with the highest rated quality and the 50% of designs with the lowest rated quality (*SI Appendix*, Fig. S2). There is suggestive evidence of a negative meta-analytic effect size in the bottom 50% of designs but not in the top 50% of designs. While the heterogeneity is statistically significant in the bottom 50% of designs, evidence of heterogeneity in the top 50% of designs is suggestive (see *SI Appendix*, section S3 for details).

We also conduct an exploratory analysis using a third analytic approach and a robustness test with clustering on the batch variable (subjects were randomized in batches of four participants to the 45 × 2 = 90 experimental treatments). These analyses yield very similar results to the ones above; see *SI Appendix*, section S3 and Tables S3 and S4.

### Moderating Effect of Common Design Choices (Not Preregistered).

An interesting question is whether (part of) the heterogeneity in effect sizes is systematically driven by certain features of the experimental designs. Yet it is not straightforward to formally address this question as no two experimental designs model competition and moral behavior exactly the same way. To venture a step in testing this conjecture, we code the 45 experimental designs along three variables, capturing potentially important characteristics of the research designs. The first variable divides the conceptualization of moral behavior into four categories: i) donations to charity (*n* = 9), ii) generosity to other participants (*n* = 9), iii) cheating/deception (*n* = 23), and iv) “other designs” that cannot be classified into any of the other three groups (*n* = 4). Two more variables concern the operationalization of the competition intervention: The first one captures whether or not there is a monetary incentive in the competition in that winning (or the rank in) the competition affects the monetary payment of the participant (“yes”: *n* = 35; “no”: *n* = 10); the second one identifies whether or not the competition is directly linked to moral behavior, i.e., whether the moral behavior as conceptualized by the teams affects the likelihood of winning (or the rank in) the competition (yes: *n* = 26; no: *n* = 19). The coding of the 45 designs along these three dimensions is shown in *SI Appendix*, Table S5; thorough descriptions of all experimental designs are provided in the RTs’ preregistrations, available at osf.io/r6anc.

To illustrate the variability in the conceptualization of moral behavior and the operationalization of competition, we exemplify the coding based on three examples. In design ACH91, participants solve as many matrices as possible in 5 min. They receive £0.10 per self-reported correct answer in both the control and the competition treatment, but in the competition treatment, participants get paid an additional £0.70 bonus if they report solving more matrices than another randomly selected participant. (Im)moral behavior is measured as the difference between self-reported and actual performance, coded as “cheating/deception” for the conceptualization of moral behavior, as yes on monetary incentives in the competition, and as yes on the moral behavior affecting the likelihood of winning the competition. The design ICP06 involves two stages. In the first stage, participants carry out a real effort task (positioning sliders at the midpoint). While there is no incentive in the control condition, the participant solving most sliders in a group of 25 players wins a £10 bonus in the competition condition. In the second stage, participants decide how much of £0.50 to donate to a charity and how much to keep for themselves, constituting the measure of moral behavior. This design is coded as “donations to charity” for the conceptualization of moral behavior, as yes on monetary incentives, and as no on moral behavior affecting the likelihood of winning the competition. The design BDQ29 also involves two stages. In the first stage, participants are matched in groups of four and participate in a real-effort task (encoding words into numbers for 5 min). In the competition treatment, participants receive symbolic medals based on their performance rank in their group, whereas in the control treatment, participants do not get any feedback on their performance or rank. In the second stage, all four players receive a 100 Taler endowment (equivalent to £0.75) and can increase or decrease the endowment of other group members by up to 30 Taler at a cost of one Taler. The average “transfer” to other players serves as the measure of moral behavior. This design is coded as “generosity to other players” for the conceptualization of moral behavior, as no on monetary incentives, and as no on moral behavior affecting the likelihood of winning the competition.

**Fig. 3.**
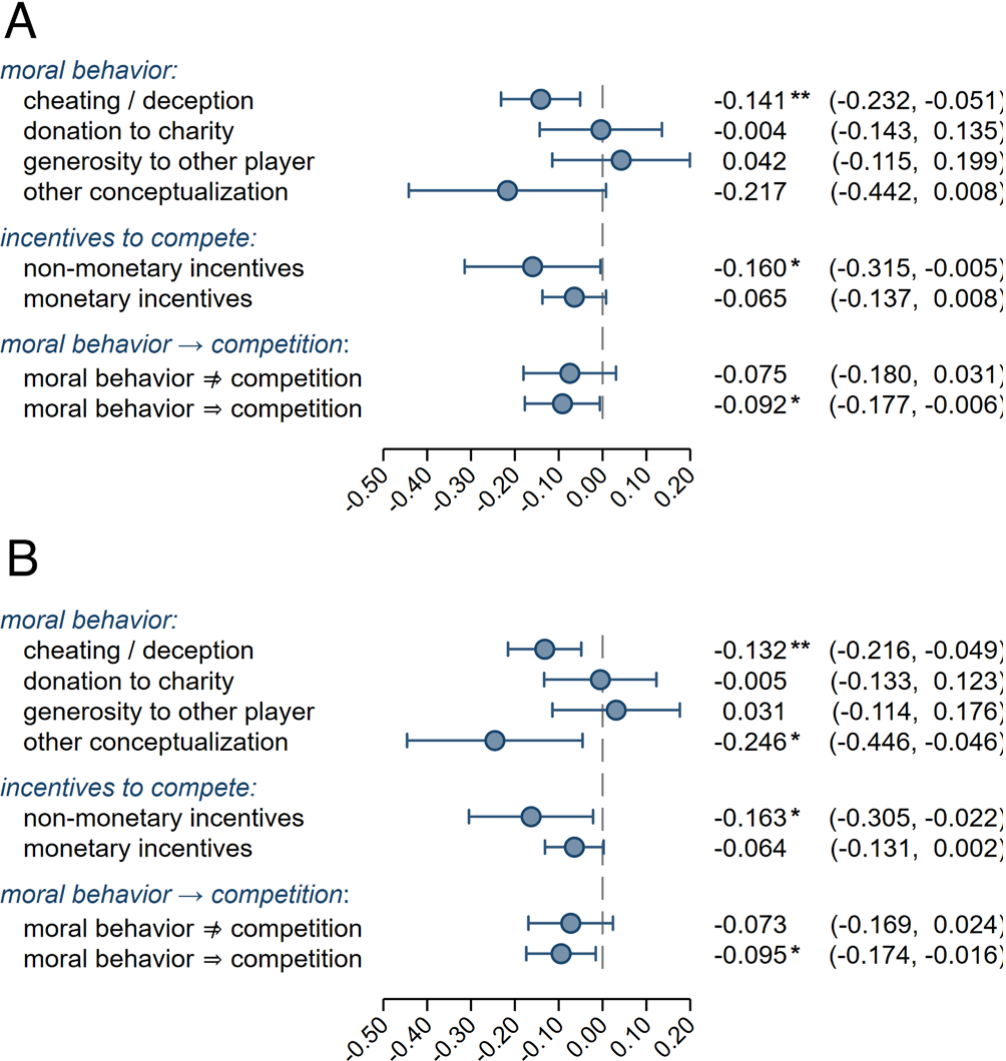
Predicted meta-analytic effect sizes in different experimental design sub-groups. (*A*) Plotted are the predicted values and 95% CIs of the meta-analytic effect size for analytic approach A, for the different conceptualizations of moral behavior and the different operationalizations of the competition intervention. The predicted values are based on the meta-regression tabulated in *SI Appendix*, Table S6, and the prediction for each design variable is carried out at the mean of the other design variables. *P < 0.05, **P < 0.005. (*B*) Plotted are the predicted values and 95% CIs of the meta-analytic effect size for analytic approach B, for the different conceptualizations of moral behavior and the different operationalizations of the competition intervention. The predicted values are based on the meta-regression tabulated in *SI Appendix*, Table S6, and the prediction for each design variable is carried out at the mean of the other design variables. *P < 0.05, **P < 0.005.

We estimate meta-regressions with the three design variables as moderators; the results are reported in *SI Appendix*, Table S6. The predicted meta-analytic effect sizes in different experimental design sub-groups based on the meta-regressions for analytic approaches A and B are depicted in Fig. 3. The three design variables are not jointly statistically significant (analytic approach A: χ^2^(5) = 7.755, *P* = 0.170; analytic approach B: χ^2^(5) = 9.312, *P* = 0.097), and they explain only 3.1% and 6.4% of the heterogeneity in analytic approaches A and B, respectively. The heterogeneity after controlling for the three design variables (*τ* = 0.182 in A; *τ* = 0.163 in B) is statistically significant for both analytic approach A (*Q*(39) = 156.9, *P* < 0.001) and analytic approach B (*Q*(39) = 136.0, *P* < 0.001) and comparable in magnitude to the main results reported above. There is suggestive evidence for the joint significance of the three moral behavior variable coefficients for analytic approach B (χ^2^(3) = 8.120, *P* = 0.044), but not for analytic approach A (χ^2^(3) = 6.869, *P* = 0.076). Joint tests of the two variables capturing different conceptualizations of competition result in *p* values exceeding the 5% suggestive evidence threshold for both analytic approach A (χ2(2) = 1.074, *P* = 0.585) and analytic approach B (χ2(2) = 1.373, *P* = 0.503). Overall, the design variables explain little of the heterogeneity, and we find no strong evidence for systematic variation in meta-analytic effects attributable to these three design characteristics. However, it should be noted that the statistical power is limited for detecting differences between subgroups of designs.

The three design variables explain 6.4% of the variation in design heterogeneity according to the above estimate for analytic approach B, which implies that the remaining design heterogeneity is explained by other design choices (“hidden moderator variables”) and/or interactions among the three modeled moderator variables. That the three modeled design choice variables only explain 6.4% of the design heterogeneity is not surprising. An interesting example to illustrate this point is the recent study by Breznau et al. (33). They conduct a multianalyst study where different RTs test the same hypothesis using the same data, but where the analysis can vary across RTs. That is, they study the heterogeneity in effect sizes due to analytical variation across RTs (while holding other sources of variation constant as the same dataset is used by all analysts). In coding ex-post a large number of analytical decisions made by analysts, Breznau et al. (33) report that only 2.6% of the variation in effect sizes can be explained by the coded analytical choices in their “prediction model.” They furthermore simulate the distribution of effect sizes based on 23 analytical decisions, yielding 2,304 unique analysis paths. They then estimate how much of the resulting simulated variation can be explained in prediction models utilizing the 23 analytical choice variables. They manage to explain at most 16% of the overall (simulated) variation with these 23 variables, although they—by definition—characterize 100% of the variation (as these variables were the only ones used to simulate the effect size distribution). The difference in explanatory power between 16% and 100% is due to nontrivial interactions between the 23 variables that can hardly be modeled. This illustrates the difficulty of predicting variation even if the included moderator variables account for 100% of the variation. The difference between the explanatory power of 2.6% in the actual data and the explanatory power of 16% in the simulated data also illustrates the difficulty of coding and measuring all relevant analytical decisions ex post (a similar challenge as trying to code and measure all the relevant design decisions ex post in our study). Further work is needed to establish which design features are crucial to explain between-study variation attributable to the variation in experimental designs. To study moderation effects, a research design in which different design variables are systematically varied while holding all other design aspects constant (rather than relying on the “natural” design variation as in the present study) would be more appropriate.

### Attrition Analysis (Not Preregistered).

Zhou and Fishbach (34) raised the important issue of selection bias due to attrition in online studies. To examine whether our results are affected by a potential attrition bias, we test whether the attrition—measured as the fraction of participants that started the experiment but did not complete it—differs between the two conditions. Particularly, we estimate a probit regression for each of the 45 designs with attrition as a function of a treatment indicator, clustering SEs on the batch randomization variable (to take into account that dropouts in a group design may imply that other group members cannot complete the experiment). These results are reported in *SI Appendix*, Table S7. The average attrition rate of 7.9% in the competition treatment and 8.6% in the control treatment do not differ statistically significantly between the treatments (paired *t* test: *t*(44) = 0.891, *P* = 0.378, *n* = 45). On average, attrition rates are quite low, but there is some variation across the designs with sizable fractions of dropouts in some designs. Among the 45 designs, there is a statistically significant difference (*P* < 0.005) in attrition for two designs and suggestive evidence (*P* < 0.05) of a difference in two designs; but note that we would expect to observe suggestive evidence for 2.25 designs by chance in 45 independent tests if the null hypothesis of no systematic attrition between treatments would be true.

To investigate whether designs involving systematically different attrition rates between treatments affect our overall conclusions, we carry out a meta-regression on an indicator variable for the four designs with statistically significant or suggestive evidence of a difference in attrition; the results are reported in *SI Appendix*, Table S8. The coefficient in the meta-regression is –0.276 (*P* = 0.010) for analytic approach A and –0.260 (*P* = 0.014) for analytic approach B, explaining 23.6% of the variation in A and 17.2% of the variation in B. The residual heterogeneity after controlling for the attrition dummy variable is still statistically significant (analytic approach A: *τ* = 0.162; *I^2^* = 70.4%; *Q*(43) = 145.4; *P* < 0.001; analytic approach B: *τ* = 0.153; *I^2^* = 68.9%; *Q*(43) = 138.4; *P* < 0.001). The meta-analytic effect size for the remaining 41 effect sizes (as predicted by the meta-regression) is –0.063 (*P* = 0.030) for analytic approach A and –0.066 (*P* = 0.029) for analytic approach B, i.e., there is suggestive evidence of a negative effect of competition on moral behavior among these 41 designs. These analyses suggest low attrition overall but indicate that differences in attrition rates may have been an issue for a few designs, potentially inflating the mean effect size and the heterogeneity somewhat.

### Limitations.

Even though our study is broad, involving multiple RTs and experimental designs, it also has important limitations. An obvious constraint is that we only include experimental designs implementable in online data collections. We cannot rule out that alternative designs implementable in the laboratory and/or the field would yield systematically different effect sizes or that effect sizes would differ for the same designs in online and laboratory settings. The more controlled environment in the laboratory may also increase standardized effect sizes by reducing the sample variance in the data. Only including online designs may have reduced the design heterogeneity compared to if we had also included laboratory and field experiments, but that is an issue to be addressed in future work. Moreover, restricting designs to online settings implies that participants were competing with strangers rather than with someone they knew, which could affect how much they care about the outcome of the competition. Attrition is also more of a problem for online experiments than in lab and field experiments. Furthermore, participants were English speakers recruited via the Prolific subject pool (which potentially involves self-selection effects), and our sample is dominated by a relatively small number of countries; we cannot rule out that results vary across populations. These design constraints limit the generalizability of our results, and especially, the meta-analytic effect size should be interpreted cautiously. Our relatively restrictive inclusion criteria for the permissible experimental designs may also imply that we underestimate the design heterogeneity compared to the existing literature investigating this research question.

## Discussion

In summary, we find an adverse effect of competition on moral behavior in the meta-analysis pooling results across the 45 experimental designs. The results are similar for analytic approaches A and B, although the evidence is statistically significant (*p* < 0.005) for B and suggestive (*P* < 0.05) for A. The estimated negative effect size is quite small with a Cohen’s *d* of about 0.1 [a Cohen’s *d* of 0.2 is typically considered a small effect size (35)]. Moreover, we find strong evidence for substantial design heterogeneity. Landy et al. (22) also estimated the heterogeneity across experimental designs for five hypotheses in psychology observing even larger heterogeneity (the average *τ* for the four hypotheses reporting effect sizes in Cohen’s *d* units was about 0.4). Both Landy et al. (22) and our study collected the data online (although using different online platforms), but there are several dimensions in which the two studies differ: the hypotheses tested, the recruitment of RTs, the standardization of the analysis across designs, and requirements imposed on the crowd-sourced experimental designs. A priori it would be expected that imposing some standardization of the experimental designs, such as requiring an “experimental economics protocol” for all designs, would lower heterogeneity, but this needs to be systematically examined in future studies.

How broadly to define the research question is an important decision to make in any study, particularly in meta-analyses. The scope for variation in experimental designs, and thus the scope for identifying design heterogeneity, is codetermined by the particular research question chosen. The research question raised in our study has been inspired by a longstanding debate in the literature, and the particular phrasing has been guided by the phrasing used in recent experimental contributions. For instance, Falk and Szech (11) formulate their research question as “does market interaction erode moral values?”—a similarly broad phrasing as we use. They do not phrase their research question as “does participating in a double-auction market make participants more likely to kill a mouse?”, but they use a very specific study design to draw conclusions as to a hypothesis involving broad concepts. Similar broadly phrased research questions have been raised in follow-up studies (12–16, 36–38), all of which use an idiosyncratic research design to draw conclusions about a generic hypothesis. The variation in experimental designs—and thus the scope for design heterogeneity—is likely to be larger in the previous literature testing comparably generic hypotheses, as the designs in our study were restricted along several dimensions (as discussed above). Ultimately, the answer to the question of how broadly to define the research question depends on what hypotheses we want our study designs to be informative about. To test whether and to which extent design heterogeneity varies with the broadness of the research question is an important question for further research. Yet, as highlighted by our study, neglecting the uncertainty due to variation in compatible study designs implies that the informativeness and conclusiveness of experimental findings based on a particular research design might be vastly overstated.

**Fig. 4.**
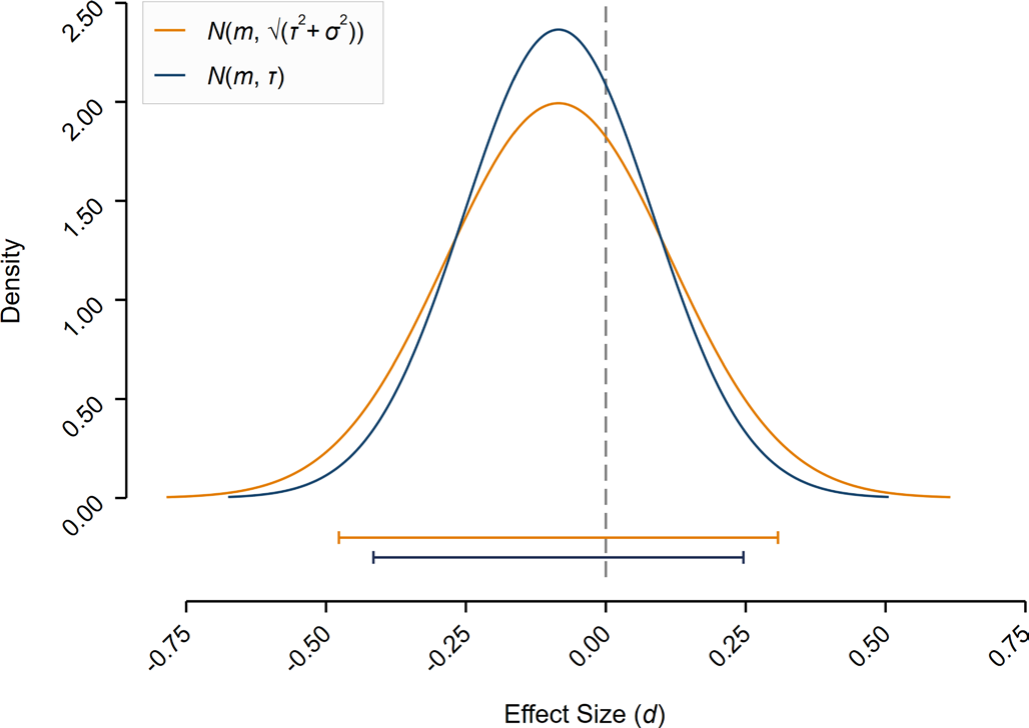
Illustration of the importance of experimental design heterogeneity. Plotted is the normal density function of the effect size distribution and the associated 95% CI of conducting a single-design study randomly drawn from the 45 experimental designs (for analytic approach B, isolating design heterogeneity). The mean of the density function is equal to the (equally weighted) mean of the 45 designs *m* = −0.085, and the variance of the density function is defined as the estimated design heterogeneity (*τ*^2^ = 0.028) plus the average sampling variance (*σ*^2^ = 0.012) of the 45 experimental designs. The 95% CI is [−0.477, 0.308], illustrating that single-design studies yield imprecise estimates if the estimated design heterogeneity is incorporated. Plotted is also the normal density function and the 95% CI based on only the design heterogeneity (*τ*^2^), providing a lower bound of the confidence interval when the sampling variance (sample size) goes to zero (infinity), illustrating that also this lower bound results in a wide confidence interval of [−0.415, 0.246]. The intervals in Fig. 4 were not preregistered and should not be interpreted as hypothesis tests, but only as an illustration of the importance of design heterogeneity.

The substantive design heterogeneity in our study reveals that generalizability based on single research designs can be limited (39–41). To illustrate this, Fig. 4 plots the expected distribution of effect sizes of randomly implementing one of the 45 designs. Taking into account the uncertainty associated with the choice of an experimental design—which is not incorporated into standard statistical testing—results in a wide 95% CI of [−0.477, 0.308], illustrating that a single design is largely uninformative about whether or not the underlying hypothesis is supported. The average sample standard error for our 45 designs is σ = 0.108, which approximately doubles if our estimated design heterogeneity is added (√(*σ*^2^ + *τ*^2^) = 0.200). The estimated *τ* of 0.169 also provides a lower bound of the standard error of a single study when the sampling variance converges to zero (i.e., for very large sample sizes). This implies a lower bound of the 95% CI of [−0.415, 0.246] and that a single-design study can at most reach 80% power to detect an effect size of 0.393 at the 0.5% level and 0.302 at the 5% level. To increase statistical power and to obtain more reliable scientific evidence, researchers would need to conduct studies based on multiple designs pooled in a meta-analysis. An essential advantage of such prospective meta-analyses is that selective reporting and publication bias, threatening the validity of standard meta-analyses, can be avoided. Moreover, the process of knowledge generation can be sped up by collecting data for many experimental designs in a single study, whereas it may otherwise take years to accumulate this amount of evidence. Our findings provide an argument for moving toward much larger data collections and more team science to improve the informativeness and generalizability of experimental research in the social sciences.

## Materials and Methods

We invited RTs to contribute experimental research designs on the question “Does competition affect moral behavior?” The goal was i) to estimate a meta-analytic effect size based on a large pool of research designs to test whether competition affects moral behavior and ii) to estimate to what extent the estimated effect size varies across alternative experimental designs. Contributing RTs proposed an experimental design and programmed their experiment; the data were collected by the project coordinators in one large online data collection at Prolific (www.prolific.co) with randomization to the different experimental designs.

Prior to starting the data collection, we preregistered a detailed PAP for the project and filed the 45 PAPs submitted by RTs (osf.io/r6anc). In *SI Appendix*, section S4, we detail any unforeseen decisions made after the preregistration, and we explicitly mention any deviations from the PAP in the main text. The preregistered hypotheses and tests were divided into four categories: i) primary hypotheses, ii) secondary hypotheses, iii) exploratory analyses, and iv) robustness tests. See *SI Appendix*, section S3, for more details about the hypotheses and tests. In all hypothesis tests, we used the preregistered thresholds of *P* < 0.005 proposed by Benjamin et al. (24) for “statistically significant” evidence and *P* < 0.05 for “suggestive” evidence. All the tests are based on two-sided *P* values.

All the experimental designs as well as the overall project received IRB approval from the University of Innsbruck (Certificate of good standing, 36/2021 for the overall project, and Certificate of good standing, 59/2021 for the 45 individual designs which were randomly selected for implementation). All participants in the Prolific data collection gave informed consent to participate. Below we describe our methods and in some cases refer to the *SI Appendix* or the PAP for further details.

### RTs and Experimental Design Requirements.

Participating RTs were required to design (and later program) an experiment with one competition and one control treatment to be administered online via Prolific with a total sample size of *n* = 400 participants for each design/experiment. Designs had to be implemented in a between-subjects setting (with, in expectation, equally sized control and treatment groups of *n* = 200 each). The rationale for having a sample size of at least 400 per study design was guided by both statistical and economic considerations. First, as we planned to have a sample of 50 RTs, we needed to make sure that the overall study was affordable in terms of resources (i.e., subject pool availability on Prolific and monetary incentives). With 50 study designs à 400 participants, we required a total sample of 20,000 participants, which we deemed feasible along both dimensions. Second, a sample of *n* = 400 is sufficiently large to obtain reasonable statistical power to detect small to medium effect sizes in terms of Cohen’s *d* units for each study design in the sample. Assuming an independent-samples *t* test, with *n*_1_ = *n*_2_ = 200, gives us 90% power to detect an effect size of *d* = 0.411 and *d* = 0.324 at the 0.5% and the 5% significance thresholds. Note, however, that the main objectives of the project were to estimate the meta-analytic effect size after pooling the data from the different study designs and to estimate the heterogeneity in results across study designs. The question of whether an individual study design yields a significant result was of minor importance to our main analysis and will only enter in terms of descriptive statistics of the fraction of designs with significant effects.

RTs were required to define a clear outcome measure of moral behavior in both treatments and a clear treatment intervention inducing competition, as well as to use monetary incentives. In the control treatment, moral behavior is measured without competition. In the competition treatment, moral behavior is measured under competition. All designs of all RTs specifically had to adhere to the following design conditions:

» Experimental participants must not be deceived at any time.
» The experiment must be based on a between-subjects treatment variation. Participants’ anonymity regarding who is interacting with whom must be preserved throughout the experiment.
» The experimental design may not involve measurements of physical state (e.g., saliva samples and blood samples) and may not involve the risk of physical or psychological harm.
» The experiment must provide clear information to subjects regarding the experiment’s duration, repetitions, interactions, and random processes (e.g., lotteries) that are relevant for participants and regarding which information is common knowledge to other (groups of) subjects. All instructions must be in English.
» The experiment must involve incentive-compatible payments for subjects that cover at least the opportunity cost of time (at the time of the experiment, Prolific implemented the requirement of £5 per hour as minimum payment); payments may not be negative. Experiments should be designed to last no longer than 15 min for participants to complete. The fixed payment (show-up fee) will be £1.30 for all experiments. The average expected bonus payment on top of this fixed payment must not exceed £1.70.
» The experiment must be designed such that it can be administered online via Prolific and such that it adheres to Prolific’s terms & conditions for researchers: Please be aware that not all kinds of interaction can plausibly be run in online experiments.
» The experimental design has to be eligible to obtain an IRB shortcut approval from the University of Innsbruck (see Appendix C in the PAP for more details; osf.io/r6anc).
» Participants will be invited from a selection of the Prolific database which is defined as follows: a) fluency in English and b) approval rate above 90%. The participants who accept the invitations will be randomly allocated to the different research designs. It is not allowed to exclude (screen out) any of the randomly assigned subjects from participating in the experiment. If RTs want to include questions in the experiment that allow them to exclude observations ex post in the RTs preregistered analysis, then this is possible but must be specified ex ante by the RT.

We invited RTs through public mailing lists (e.g., ESA and JDM lists), social media (e.g., Twitter), and sent out direct emails to colleagues with the invitation to participate and/or advertise the study within their network (see Appendix D in the PAP for the invitation letter; osf.io/r6anc). The recruitment of RTs involved the following two steps.

In the first step, potential RTs had to fill out a short application form with background information (see Appendix A in the PAP; osf.io/r6anc). Based on the form, we screened out RTs according to the eligibility conditions and invited the RTs that passed the screening to submit a proposed research design. Participants could only be a member of one RT and each RT could only submit one design. Each RT could consist of a maximum of two members and at least one member had to hold a PhD in Economics, Psychology, or a related field. To make sure that RTs had previous experience with designing experiments, at least one member needed to have published at least one experimental paper (published, accepted for publication, or published as a working paper/preprint). RTs also had to justify why they considered themselves eligible for the study in the application form. In the second step, RTs that passed the initial screening submitted their research designs according to a preregistration template (see Appendix B in the PAP; osf.io/r6anc). In this preregistration, RTs were required to prespecify the measure of moral behavior, the control and the competition treatment, as well as their preregistered analyses.

To select the participating RTs out of the submitted experimental designs, we first prescreened all submitted proposals to make sure they conformed with the RT constraints as specified above (eligible submissions). We decided to include at most 50 research designs and as we received 95 designs, we randomly selected 50 designs out of those for inclusion in the study. To be as transparent as possible, we preregistered the Stata script used to randomly select the 50 designs (see Appendix F in the PAP; osf.io/r6anc). After the RTs were selected for participation, they had to program the experimental software for their own designs and were responsible for hosting the experiment online. The project coordinators recruited participants for the experiment via Prolific and paid for the experiments. In the process of implementing the experiments, the number of designs was reduced from 50 to 45: four RTs did not submit the code/software to run the experiment and were thus excluded; for one RT, we discovered during the piloting that their design could not be implemented using Prolific (henceforth “pilot dropout”). We carried out two pilot sessions with around 360 participants each (i.e., in each pilot aiming for four participants in each of the two treatments of all 45 implemented designs) to ensure that the software and the randomization of participants to the various experiments were running properly; the pilot data are not included in the dataset used in the study.

RTs whose designs were included in the project (and the pilot dropout) were invited to be coauthors on the paper that results from the project. The link between individual researchers and the individual designs is not revealed in the paper, but as they are coauthors, they are not fully anonymous. The 45 RTs consisted of 86 researchers, of which 17.4% did not hold a PhD, 43.0% held a PhD in economics, 26.7% held a PhD in psychology, 5.8% held a PhD in behavioral sciences, 4.7% held a PhD in business studies, and 2.3% held a PhD in finance. Of the researchers, 75.6% were affiliated with a research institution in Europe, 19.8% in North America, 3.5% in Asia, and 1.2% in Australia.

### Data Collection and Randomization of Participants to the Different Designs.

In recruiting and assigning experimental participants to the 45 different designs, we used the following procedures:

1. There was one common Prolific study which was set up by the project coordinators and which directed participants to a common welcome screen.
2. At the common welcome screen, all participants were required to sign a captcha (to rule out bots), to provide informed consent, and to complete a common attention check question (see Addendum Appendix 1 in the addendum to the PAP for these screens; osf.io/r6anc).
3. After that, participants were randomly redirected to the 90 (45 designs × 2 treatments) individual treatments in batches of four participants, i.e., four participants in a row were redirected to the same treatment of the same experiment/design. This was to avoid problems with experiments involving real-time interactions in groups of two or four participants (with randomization at the individual level and 90 treatments, it could have been a long waiting period until there are two or four participants to form one group that plays the game simultaneously, potentially leading to a high fraction of dropouts during the waiting period). We randomly assigned the first 45 × 2 = 90 batches of four participants (i.e., the first 360 participants) to each of the 90 treatments (i.e., one batch per treatment) and so on for the next 90 batches, etc. This procedure implies that it was only possible to use groups of either two or four participants simultaneously interacting in the same group during the experiment (and, of course, designs without any groups or designs with groups defined ex post). For a few experiments (JTI38, LGT85, PKY70, and ZZS69), this led to some minor adjustments in the design changing group sizes from six, five, or three to four or two or to ex post matching of groups.
4. We collected the data in ten time slots during the 2 wk from January 17 to January 28, 2022, with one slot per day starting at 2 pm (GMT) from Monday to Friday in each week. The aim was to collect 1,800 “completed observations” in each time slot, where a completed observation is defined as a participant that submits a valid and correct Prolific completion code. We opened the data collection at each time slot at 2 pm (GMT) and continued until we reached 1,800 completed observations or until 4 pm (GMT). After the end of the tenth time slot, we counted the total number of completed observations and continued the data collection the same day in increments of 360 (90 × 4) completed observations until we reached at least 18,000 “completed observations” (implying at least an average of 400 completed observations per design). In the end, we reached 18,123 completed observations. We used the procedure with multiple time slots to reduce the risk of technical problems, which would have been more likely if we would have administered the experiment with more than 18,000 participants at the same time.

Individual characteristics of the participants who completed the experiment do not differ systematically between the designs. Particularly, we estimate linear regressions of each characteristic on research team fixed effects and test whether the fixed effects are jointly statistically significant. We carry out this test for the following characteristics: age, gender, Prolific score, fraction fully employed, fraction of students, fraction with first language English, fraction from the United Kingdom, fraction from the United States, and fraction from South Africa. We find no evidence of a statistically significant difference between the 45 designs for any of these characteristics (*P* > 0.05 for all these tests); see *SI Appendix*, Fig. S1 for details. These analyses were not preregistered.

### Peer Assessment.

Before the Prolific data collection, participating RTs were asked to assess each other’s designs anonymously (based on the preregistration template submitted by each RT). In particular, each RT was asked to assess ten other randomly selected designs and rate them on a Likert scale from 0 to 10 based on the following question:

*To what extent does this design, within the design conditions defined above, provide an informative test of the research question: “Does competition affect moral behavior?”*

- 0 (not at all informative)
- 1
- …
- 9
- 10 (extremely informative)

RTs consisting of two members were asked to coordinate and required to submit one rating per design only. The designs to be assessed by each RT were randomly determined. We demean the RTs’ quality ratings (i.e., we subtract the mean rating of an RT from every rating of this RT) before we estimate the average peer assessment score for each design. All analyses are based on this demeaned score.

### Analytic Approaches.

We estimate our results for two analytic approaches A and B. For both analytic approaches in any of the analyses, we only include “completed observations,” defined as participants who submitted a valid and correct Prolific completion code. Any participant who was randomized into one of the designs was also excluded from participating in the study again, irrespective of whether the participant completed the study (submitted a Prolific completion code) or whether the participant dropped out before completion. We tried to rule out repeated participation, but 709 participants still managed to participate in the study again. We excluded all subsequent observations for these 709 participants in both analytic approaches A and B. Specifically, the first observation of the participant was included if the participant submitted a Prolific completion code for that observation, but any subsequent observations from that participant were excluded. The requirement that participants could only participate once in the study was preregistered. Below we describe analytic approaches A and B:

A.  Standardizing effect sizes across RTs: For each RT study, we estimate the effect size and standard error according to the proposed/preregistered analysis and econometric specification that has been preferred by the RT as the best approach to answer the research question (see the preregistration template in Appendix B in the PAP and the 45 preregistered PAPs submitted by the RTs; osf.io/r6anc). In case the specification of the statistical analysis was not sufficiently clear, we contacted RTs individually to resolve any uncertainties. The only condition for the analysis was that the RTs had to prespecify an ordinary least squares regression for their chosen main analysis, with moral behavior as the dependent variable and an indicator variable for the competition treatment. To be able to conduct a meta-analysis based on the results for analytic approach A, we standardize the treatment effects across RTs using Cohen’s *d*.
B.  Standardizing the analytic approach across RTs (in addition to standardizing effect sizes across RTs): To remove as much of the analytical variation across RTs as possible, we estimate the meta-analytic effect size and between-study heterogeneity based on a standardized analytical approach across the RT designs. In this standardization, we use the same ordinary least squares regression as in analytic approach A but with some adaptations as described further in *SI Appendix*, section S2 (as for analytic approach A, we also standardize the reported treatment effects across RTs using Cohen’s *d*).

The conversion of effect sizes to Cohen’s *d* is described in *SI Appendix*, section S1. We estimate two separate meta-analyses: one based on the effect sizes from the analyses proposed by the RTs (as described in analytic approach A) and one based on the effect sizes from the standardized analytical approach as described in analytic approach B. The RTs delivered the raw data from their design to the project coordinators and information about the coding of variables (i.e., a code book); the project coordinators then estimated the effect size and standard error for each experimental design for both analytic approaches A and B. The result of analytic approach A was shared with the RTs, and they were asked to assess the plausibility of the result (in terms of the magnitude and the sign of the effect). RTs were not asked to reproduce the exact results as they were not informed about which of the participants recorded in their dataset eventually provided a valid Prolific completion code.

## Supplementary Material

Appendix 01 (PDF)

## Data Availability

The data and analysis code are available at the project’s OSF repository (osf.io/r6anc). The mean and standard error resulting from each experimental design for the different analytical approaches and the peer rating for each experimental design are tabulated in *SI Appendix*, Tables S1–S4. The PAP of the overall project, the preregistration of each experimental design, the materials submitted by RTs to collect data for each experimental design, and the individual level data for each experimental design are available at the project’s OSF repository (osf.io/r6anc). Anonymized pre-analysis plan, experimental design proposals, materials, data and codes data have been deposited in OSF Open Science Framework osf.io/r6anc.
