# Supplementary material for: Competition and moral behavior: A meta-analysis of forty-five crowd-sourced experimental designs
Source: Proc Natl Acad Sci U S A. 2023 May 30;120(23):e2215572120. doi: 10.1073/pnas.2215572120 (PMC10266008; doi:10.1073/pnas.2215572120)
Supplement: Supplementary file 1 — Appendix 01 (PDF) [file pnas.2215572120.sapp.pdf]

PNAS

Supporting Information for

Competition and moral behavior: A meta-analysis of forty-five crowd-sourced experimental designs.

Christoph Huber, Anna Dreber, Jürgen Huber, Magnus Johannesson, Michael Kirchler, Utz Weitzel, Miguel Abellán, Xeniya Adayeva, Fehime Ceren Ay, Kai Barron, Zachariah Berry, Werner Bönte, Katharina Brütt, Muhammed Bulutay, Pol Campos-Mercade, Eric Cardella, Maria Almudena Claassen, Gert Cornelissen, Ian G. J. Dawson, Joyce Delnoij, Elif E. Demiral, Eugen Dimant, Johannes Theodor Doerflinger, Malte Dold, Cécile Emery, Lenka Fiala, Susann Fiedler, Eleonora Freddi, Tilman Fries, Agata Gasiorowska, Ulrich Glogowsky, Paul M. Gorny, Jeremy David Gretton, Antonia Grohmann, Sebastian Hafenbrädl, Michel Handgraaf, Yaniv Hanoch, Einav Hart, Max Hennig, Stanton Hudja, Mandy Hütter, Kyle Hyndman, Konstantinos Ioannidis, Ozan Isler, Sabrina Jeworrek, Daniel Jolles, Marie Juanchich, Raghabendra Pratap KC, Menusch Khadjavi, Tamar Kugler, Shuwen Li, Brian Lucas, Vincent Mak, Mario Mechtel, Christoph Merkle, Ethan Andrew Meyers, Johanna Mollerstrom, Alexander Nesterov, Levent Neyse, Petra Nieken, Anne-Marie Nussberger, Helena Palumbo, Kim Peters, Angelo Pirrone, Xiangdong Qin, Rima Maria Rahal, Holger Rau, Johannes Rincke, Piero Ronzani, Yefim Roth, Ali Seyhun Saral, Jan Schmitz, Florian Schneider, Arthur Schram, Simeon Schudy, Maurice E. Schweitzer, Christiane Schwieren, Irene Scopelliti, Miroslav Sirota, Joep Sonnemans, Ivan Soraperra, Lisa Spantig, Ivo Steimanis, Janina Steinmetz, Sigrid Suetens, Andriana Theodoropoulou, Diemo Urbig, Tobias Vorlaufer, Joschka Waibel, Daniel Woods, Ofir Yakobi, Onurcan Yilmaz, Tomasz Zaleskiewicz, Stefan Zeisberger, Felix Holzmeister

To whom correspondence should be addressed:

Felix Holzmeister, E-Mail: felix.holzmeister@uibk.ac.at

This PDF file includes:

SI Methods
Tables S1–S8
Figures S1–S2

SI Methods

Below we provide further details about *(i)* the conversion of effect sizes to Cohen's *d*, *(ii)* the standardization of the analyses across RTs, *(iii)* the hypotheses addressed and the tests used, and *(iv)* the pre-registration and any deviations thereof. We also refer to the pre-analysis plan (PAP) posted at OSF (osf.io/r6anc) for some further details. The project was accompanied by a dedicated website: www.manydesigns.online.

1. Converting effect sizes to Cohen's *d*

The dependent variable (the outcome measure) in the analyses by the RTs was moral behavior and the main independent variable was an indicator variable for the two experimental treatments (the control group coded as 0 and the competition group coded as 1). As RTs were expected to use different measures of moral behavior, we needed to standardize the treatment effects across RTs to be able to conduct a meta-analysis. We therefore convert all treatment effects to Cohen's *d* such that they are measured in terms of standard deviation (*SD*) units of the outcome variable. To convert effect sizes to Cohen's *d* and to determine the corresponding standard error (*SE*) we used the following formulas:

Cohen's *d* = effect size / pooled *SD*

SE of Cohen's *d* = *SE* of effect size / pooled *SD*

where the effect size is the estimated treatment effect before standardization, i.e., the coefficient estimate of the treatment indicator variable in the ordinary least squares regression. We align the signs of the effect size measures across teams such that a positive (negative) effect corresponds to an increase (decrease) in moral behavior due to competition. The standard error of the effect size is the standard error of the treatment indicator variable in the ordinary least squares regression. The pooled *SD* was defined as:

$$\text{Pooled } SD = [((n_1 - 1) \cdot var_1 + (n_2 - 1) \cdot var_2) / (n_1 + n_2 - 2)]^{0.5}$$

where var_1 (var_2) is the variance of the outcome variable and n_1 (n_2) is the sample size in the control (treatment) group. We estimated the pooled *SD* (var_1, var_2, n_1, and n_2) based on the sample used in the analysis where we standardized the analytical approach across RTs (analytic approach B); this estimate of the pooled *SD* was used in all conversions of effect sizes to Cohen's *d* for that RT (i.e., also for analytic approach A) to ensure that any differences in Cohen's *d* effect sizes between analytic approaches A and B were not driven by differences in the *SD* used in the conversion of effect sizes to Cohen's *d*.

2. Standardizing the analytic approach across RTs

In analytic approach A (as outlined in the PAP; see osf.io/r6anc), the effect size of the RT is based on the coefficient estimate of the competition treatment indicator variable in the ordinary least squares regression as specified by the RT in their pre-registration, using the exclusion criteria defined by the RTs. In analytic approach B, we standardize the analyses based on the regression model in analytic approach A, but with four possible adaptations:

1. We remove all control variables from the regression such that the only independent variable in the ordinary least squares regression was the indicator variable for the competition treatment.
2. We generally include all observations with data on the dependent variable; as long as this is a "completed observation" as defined above (i.e., as long as the participant submitted a valid and correct Prolific completion code). The exception to this are data exclusions in some of

the designs due to that some participants were assigned a passive role in the experiment or their decisions do not enter the outcome measure (e.g., the dependent variable in one of the designs is based on the back-transfers by trustees in a trust game; if the trustor transferred zero in the first stage, the trustee did not have any money to return and the back-transfer could only be zero). All pre-registered data exclusions in analytical approach B are listed in the PAP (see Addendum Appendix 2 in the addendum to the PAP, osf.io/r6anc).

3. We use the individual as the level of observation and only include one observation per individual. This was already the case in all analyses in analytic approach A except for one design (`GWP43`). In this design (`GWP43`), the analysis specified by the RT (as used in analytic approach A) includes three morality ratings per subject as three separate observations per subject; in analytic approach B we instead use the average of the three observations as the dependent variable (and this average is also used to estimate the pooled *SD* to convert the effect size to Cohen's *d* for this study).
4. We estimate the ordinary least squares regression for each RT with robust standard errors (irrespectively of whether this was included in the analysis proposed by the RT in analytic approach A).

3. Hypotheses and tests

In all hypothesis tests we used the threshold of $p < 0.005$ proposed by Benjamin et al.[23] for "statistically significant" evidence, and $p < 0.05$ for "suggestive" evidence. All the tests are based on two-sided *p*-values. The choice of the significance threshold was pre-registered (see osf.io/r6anc).

We test our pre-registered hypotheses with the pre-registered tests/measures mentioned below. The pre-registered hypotheses and tests were divided into four categories: *(i)* primary hypotheses, *(ii)* secondary hypotheses, *(iii)* exploratory analyses, and *(iv)* robustness tests. Any deviations from the pre-registration are detailed in section 8. During the review process, the editor and two anonymous reviewers suggested further exploratory analyses and robustness tests. We are grateful for the suggested amendments and have added the following two paragraphs of not pre-registered results in the main text: Moderating effect of common design choices; Attrition analysis (including also Figure 3 and Tables S5–S8). We have also added the not pre-registered analyses in Figure S1, testing if there is a statistically significant difference in individual characteristics (among participants that completed the experiment) between the 45 experimental designs (these results are referred to in the Introduction section and in the Materials and Methods section of the main text).

Primary Hypothesis 1: Competition affects moral behavior.

<u>Primary hypothesis test 1A:</u> Meta-analytic effect size based on analytic approach A (standardizing the effect sizes across RTs; but not standardizing the analytic approach across RTs) .

<u>Primary hypothesis test 1B:</u> Meta-analytic effect size based on analytic approach B (standardizing the effect sizes and the analytic approach across RTs).

We test this hypothesis in a random effects meta-analysis using the DerSimonian-Laird model[24]. We report the *p*-value of the meta-analytic effect (*z*-test based on the meta-analytic effect size and its standard error) and the 95% confidence interval. We consider the meta-analytic effect size estimated in primary hypothesis 1B to be our preferred meta-analytic effect size, because it standardizes the analytic approach across RTs in a way to as much as possible avoid bias due to the use of exclusion criteria that may cause selection bias. On the other hand, the meta-analytic effect size and its associated standard error in primary hypothesis test 1A incorporates both design heterogeneity and analytical heterogeneity, and the estimated standard error could be argued to

be a better estimate of the uncertainty of the estimated meta-analytic effect size (it might be argued also that the pooled effect size across analytical approaches in analytic approach A is a more representative estimate of the meta-analytic mean effect size).

In addition to the two tests of primary hypothesis 1, we also report significance tests of the results for each RT. We report the *p*-value of the hypothesis test of each RT and a 95% confidence interval of the regression coefficient (after converting effect sizes and standard errors to Cohen's *d* to make them comparable across RTs). This is done for both analytic approaches A and B. We do not view these results for each RT as hypotheses tests, but part of the descriptive results we report. These confidence intervals are shown in Figure 1 in the main text together with the random effects meta-analysis results of primary hypothesis 1A and 1B; the exact confidence intervals and *p*-values are reported in Tables S1–S2.

Primary Hypothesis 2: There is heterogeneity in the estimated effect size across RTs.

<u>Primary hypothesis test 2A:</u> Heterogeneity in the meta-analytic effect size based on analytic approach A (standardizing the effect sizes across RTs; but not standardizing the analytic approach across RTs) .

<u>Primary hypothesis test 2B:</u> Heterogeneity in meta-analytic effect size based on analytic approach B (standardizing the effect sizes and the analytic approach across RTs).

This test was based on the random effects meta-analyses used to test primary hypotheses 1A and 1B above. We report the estimates of three different measures of heterogeneity (Cochran's Q, τ, and I^2). We report Q and the associated *p*-value (as based on the χ^2-Test), which is commonly used to test the null hypothesis of homogeneity across effect sizes in random-effects meta-analyses. This *p*-value is used as the hypothesis test criterion for primary hypotheses 2A and 2B. For τ and I^2, we report 95% confidence intervals. τ is the estimate of the standard deviation of the true meta-analytic effect size (i.e., it captures the variation in effect sizes across RT designs over and above sampling variation) and is our preferred measure of heterogeneity and our main outcome measure in the study alongside the meta-analytic effect size estimated in primary hypothesis 1. I^2 measures the percentage of variation across studies that is due to heterogeneity rather than chance. It is difficult to interpret I^2 as an absolute measure of heterogeneity as it depends on the sampling variation, and therefore we prefer τ as a measure of heterogeneity. But we also report I^2 and its 95% confidence interval as a secondary heterogeneity measure as it is commonly reported in random effects meta-analysis. These heterogeneity results and tests of primary hypotheses 1A and 1B are reported in the main text.

The test of primary hypothesis 1B is a test of whether there is design heterogeneity as only the design varies between RTs in analytic approach B. For analytic approach A both the design and the analysis can vary between RTs, and we therefore view the test of primary hypothesis 1A as a test of if there is "design heterogeneity and/or analytical heterogeneity."

Secondary hypothesis 1: Effect size estimates across teams vary systematically with the mean peer assessments.

<u>Secondary hypothesis test 1A:</u> Effect of peer assessments on effect size estimates across RTs based on analytic approach A (standardizing the effect sizes across RTs; but not standardizing the analytic approach across RTs) .

<u>Secondary hypothesis test 1B:</u> Effect of peer assessments on effect size estimates across RTs based on analytic approach B (standardizing the effect sizes and the analytic approach across RTs).

Based on the random effects meta-analyses in primary hypothesis 1A and 1B above, we conduct meta regressions with the average peer assessments entering as a moderator variable (using the demeaned peer evaluator score as described above). This analysis was carried out for both analytic approaches A and B. Figure 2 in the main text plots the relationship between the mean rating of design quality and the estimated effect sizes (for both analytic approaches A and B) and reports the tests of secondary hypothesis 1A and 1B.

Secondary hypothesis 2: There is heterogeneity in the estimated effect size across RTs after controlling for the heterogeneity due to mean peer assessment.

Secondary hypothesis test 2A: Heterogeneity in the meta-analytic effect size based on analytic approach A (standardizing the effect sizes across RTs; but not standardizing the analytic approach across RTs) after controlling for the heterogeneity due to mean peer assessment.

Secondary hypothesis test 2B: Heterogeneity in meta-analytic effect size based on analytic approach B (standardizing the effect sizes and the analytic approach across RTs) after controlling for the heterogeneity due to mean peer assessment.

This hypothesis test was based on the meta-regressions used to test secondary hypotheses 1A and 1B and we estimated Q, τ, and I^2 for the residual heterogeneity. These residual heterogeneity measures, thus, refer to how much of heterogeneity remains after adjusting for the effect of the moderator variable (the average peer assessment score). We report the p-value associated with Cochran's Q and 95% CIs for τ and I^2. The p-value of Cochran's Q is used as the criterion for the hypothesis test of secondary hypotheses 2A and 2B. The heterogeneity results and the tests of secondary hypotheses 2A and 2B are reported in the main text. Moreover, we report the R^2 of the residual heterogeneity as a measure of how much of the variation (i.e., as measured by τ) was explained by the moderator. The heterogeneity is statistically significant after controlling for the rated quality for both analytic approaches A ($Q(43) = 179.3$, $p < 0.001$) and B ($Q(43) = 159.3$, $p < 0.001$). For analytic approach A, the estimated τ, after controlling for rated quality, is 0.187 (95% CI [0.149, 0.264]); and 76.0% (95% CI [66.9, 86.4]) of the variation in results across research designs is explained by heterogeneity. For analytic approach B, the estimated τ, after controlling for rated quality, is 0.170 (95% CI [0.142, 0.259]); and 73.0% (95% CI [65.5, 86.3]) of the variation in results across research designs is explained by design heterogeneity. The residual $R^2 = 0.000$ for both analytic approaches A and B.

The test of secondary hypothesis 2B is a test whether there is design heterogeneity after controlling for the heterogeneity due to mean peer assessment. For analytic approach A, both the design and the analysis can vary between RTs, and we therefore view the test of secondary hypothesis 2A as a test of if there is "design heterogeneity and/or analytical heterogeneity" after controlling for the heterogeneity due to mean peer assessment.

Exploratory analyses

In order to better understand what is driving any potential differences in the results above for analytic approaches A and B, we also estimate the results above for a third analytic approach (C). In this analysis, we use our standardized analysis across RTs based on the same exclusion criteria as used in analytic approach A (i.e., we implement our standardized analysis in analytic approach B using the exact same samples as included in the analyses for analytic approach A). This additional analytic approach has been included to shed light on how important the variation in exclusion criteria used by RTs is for the results and for explaining any differences in results between analytic approaches A and B. As the results for analytic approaches A and B turned out to be very similar, it is not surprising that the results for analytic approach C are also very similar with a meta-analytic effect size of $d = -0.084$ (95% CI [−0.143, −0.025], $p = 0.005$) and statistically significant

heterogeneity ($\tau = 0.171$, 95% CI [0.140, 0.254]; $Q(44) = 159.3$, $p < 0.001$; $I^2 = 72.4\%$, 95% CI [63.6, 85.3]). See also Table S3 with the results for each of the 45 designs. As for analytic approaches A and B, peer quality ratings (secondary hypothesis 1) are not systematically related to the individual studies' effect size estimates for analytic approach C in a meta regression ($b = 0.030$, $se = 0.031$, $p = 0.329$; $R^2 = 0.000$). For analytic approach C, the heterogeneity (secondary hypothesis 2) is still statistically significant after controlling for the rated quality ($Q(43) = 158.0$, $p < 0.001$); the estimated τ after controlling for rated quality is 0.173 (95% CI [0.142, 0.259]) and 72.8% (95% CI [64.4, 85.7]) of the variation in results across research designs is explained by design heterogeneity.

In an additional exploratory analysis, we estimate the results of primary hypothesis tests 1A, 1B and 2A and 2B for the 50% of RT designs with the highest peer assessment rating and the 50% of RT designs with the lowest peer assessment rating. This analysis has been done to shed additional light on to what extent our results are driven by experimental designs considered to be of low quality by peers. For analytic approach A, the meta-analytic effect size is $d = -0.047$ (95% CI [−0.109, 0.015], $p = 0.137$, $n = 22$) in the top-rated designs and $d = -0.128$ (95% CI [−0.235, −0.022], $p = 0.018$, $n = 23$) in the bottom rated designs. The standard deviation of the true effect size across experimental designs (τ) is 0.104 (95% CI [0.042, 0.187]; $Q(21) = 41.6$, $p = 0.005$, $n = 22$) in the top rated designs and 0.238 (95% CI [0.177, 0.366]; $Q(22) = 135.6$, $p < 0.001$, $n = 23$) in the bottom rated designs and the fraction of the variation explained by heterogeneity (I^2) is 49.6% (95% CI [14.1, 76.2]) in the top rated designs and 83.8% (95% CI [74.1, 92.4]) in the bottom rated designs. For analytic approach B, the meta-analytic effect size is $d = -0.043$ (95% CI [−0.104, 0.017], $p = 0.159$, $n = 22$) in the top-rated designs and $d = -0.132$ (95% CI [−0.228, −0.035], $p = 0.008$, $n = 22$) in the bottom rated designs. The standard deviation of the true effect size across experimental designs (τ) is 0.098 (95% CI [0.035, 0.184]; $Q(21) = 39.4$, $p = 0.009$, $n = 22$) in the top rated designs and 0.212 (95% CI [0.169, 0.358]; $Q(22) = 117.0$, $p < 0.001$, $n = 23$) in the bottom rated designs and the fraction of the variation explained by heterogeneity (I^2) is 46.7% (95% CI [10.0, 75.6]) in the top rated designs and 81.2% (95% CI [73.2, 92.5]) in the bottom rated designs. These results are illustrated graphically in Figure 3 in the main text.

Robustness tests

As a pre-registered robustness test, we also re-estimate all our results for analytic approach B with clustering on the batch variable (of four participants). As the randomization of participants to the 45×2 = 90 different treatments (see Supporting Information, section 2) was carried out on the batch level, it could be argued that the standard errors should be clustered at the batch level. These results are reported in the main text and in Table S4. Clustering on the batch level has little impact on our results, resulting in a meta-analytic effect size of $d = -0.080$ (95% CI [−0.133, −0.026]; $p = 0.004$) and statistically significant heterogeneity ($\tau = 0.150$, 95% CI [0.125, 0.244]; $Q(44) = 136.8$, $p < 0.001$; $I^2 = 67.8\%$, 95% CI [59.5, 84.8]). See also Table S4 with the results for each of the 45 designs. Peer quality ratings do not systematically moderate the individual studies' effect size estimates for the robustness analysis with clustering on the batch level in a meta regression ($b = 0.030$, $se = 0.028$, $p = 0.286$; $R^2 = 0.000$). The heterogeneity is still statistically significant after controlling for the rated quality ($Q(43) = 135.2$, $p < 0.001$); the estimated τ after controlling for rated quality is 0.152 (95% CI [0.128, 0.249]) and 68.2% (95% CI [60.4, 85.3]) of the variation in results across research designs is explained by design heterogeneity. As per our PAP, we also report the meta-analytic effect size and heterogeneity estimates separately for the top and bottom 50% of the sample as based on the peer quality assessments: The meta-analytic effect size is $d = -0.040$ (95% CI [−0.101, 0.020], $p = 0.194$, $n = 22$) in the top rated designs and $d = -0.123$ (95% CI [−0.210, −0.036], $p = 0.006$, $n = 23$) in the bottom rated designs. The standard deviation of the true effect size across experimental designs (τ) is 0.099 (95% CI [0.037, 0.186]; $Q(21) = 39.8$, $p = 0.008$, $n = 22$) in the top rated designs and 0.184 (95% CI [0.151, 0.348];

$Q(22) = 92.9$, $p < 0.001$, $n = 23$) in the bottom rated designs and the fraction of the variation explained by heterogeneity (I^2) is 47.2% (95% CI [11.1, 76.0]) in the top rated designs and 76.3% (95% CI [68.3, 92.0]) in the bottom rated designs.

4. Pre-registration

We pre-registered an overall analysis plan (PAP) for the project in three steps. The first part with the overall design was pre-registered at OSF (osf.io/zhqfr) on April 28, 2021, before we sent out the first invitation to participate in the project on April 29, 2021. We then, as planned and mentioned in the overall PAP, added a first addendum to this pre-analysis plan after the RTs had submitted their designs and before collecting any data in the project (osf.io/te8gb). In this first addendum, we provide the exact hypotheses, tests, and details about standardizing effect sizes and analyses across RTs. Finally, we added a short second addendum to the pre-analysis plan clarifying several issues about the data collection and analysis that arose during piloting the logistics of the Prolific data collection; this was done prior to starting the Prolific data collection (osf.io/jyndw).

In the initial PAP, we wrote that we will randomly select 42 designs out of the eligible submissions. As we received more submissions than expected (we received 95 eligible submissions), we decided to randomly select 50 designs (RTs) instead of 42; this was pre-registered as part of the first addendum to the PAP prior to starting the data collection. The 45 RT analysis plans that we eventually used for analytic approach A were also pre-registered before the Prolific data collection.

Eventually, we had to make several decisions ex post after the data collection that were not included in the PAP. All decisions not explicitly mentioned in the PAP (or one of the two addenda) are detailed below:

One design (ICP06) included an interaction between the treatment dummy variable and a control variable (measuring the score of a real effort task) in the analysis specification that was pre-registered by the RT for analytic approach A. As the coefficient of the treatment dummy variable (supposed to measure the treatment effect) does not measure the treatment effect when the interaction is included in the regression model, we changed this analysis to a regression without the interaction and the control variable. The project coordinators should have noted the problem with the interaction specification in the screening of the RT design and should have asked the RT to change the analytic specification; but the failure to do so resulted in this ex-post decision to exclude the interaction from the analysis specified by the RT (note that this change only affects analytic approach A, but not analytic approach B).

One design (PCS27) did not include any bonus payments although the use of incentive compatible payments was one of the design conditions for inclusion in the study (see Supporting Information, section 1). Accordingly, this design should not have been included in the study from the outset, but unfortunately the project coordinators failed to realize that the study did not include any bonus payments until after the Prolific data collection had been completed. As it still (incorrectly) passed our screening and the data was collected, we include this design in the analyses. Our results are not sensitive to this decision. The effect size of this study was −0.023 (95% CI [−0.213, 0.167], $p = 0.812$) for both analytic approaches A and B. If it is excluded from the analysis, the meta-analytic effect size is −0.086 (95% CI [−0.150, 0.022], $p = 0.008$) for analytic approach A and −0.087 (95% CI [−0.147, −0.028], $p = 0.004$) for analytic approach B; the estimated τ is 0.188 (95% CI [0.149, 0.264]) for analytic approach A and 0.171 (95% CI [0.142, 0.258]) for analytic approach B, and the heterogeneity test is still statistically significant ($Q(43) = 180.6$, $p < 0.001$ for analytic approach A and $Q(43) = 161.1$, $p < 0.001$ for analytic approach B).

One RT (XZK69) used two initial comprehension questions to exclude participants from participating in the experiment (one question about if they are fluent in English and one reading comprehension

check; note also that we already from the beginning only invited participants hat are fluent in English from the Prolific database). To exclude (screen out) any of the randomly assigned subjects from participating in the experiment was not allowed according to our design conditions (see Supporting Information, section 1). These two exclusion criteria should thus not have been included in this design, but it was not possible for the organizers to discover this as the RT hosted the software of the design and did not mention in the pre-registration template that these exclusion criteria would be used to screen out participants from participating in the experiment; the RT only mentioned that they would include three attention check questions to exclude participants and we interpreted this as these exclusions would be applied after completing the experiment. As this was not discovered before the data was collected, we include this design in the analyses based on these two exclusion criteria; but only two participants were excluded from participating due to these two exclusion criteria (and these participants are by definition excluded in analytic approaches A, B and C; in line with our pre-registration the third attention check question used to exclude participants that completed the experiment is also applied in analytic approaches A and C, but not in B). Our results are not sensitive to the results of this design. The effect size of this study was −0.044 (95% CI [−0.265, 0.177], $p = 0.695$) for analytic approach A and −0.048 (95% CI [−0.251, 0.154], $p = 0.639$) for analytic approach B. If it is excluded from the analysis, the meta-analytic effect size is −0.086 (95% CI [−0.149, −0.022], $p = 0.009$) for analytic approach A and −0.086 (95% CI [−0.146, −0.027], $p = 0.004$) for analytic approach B; the estimated τ is 0.187 (95% CI [0.149, 0.264]) for analytic approach A and 0.171 (95% CI [0.142, 0.259]) for analytic approach B, and the heterogeneity test is still statistically significant ($Q(43) = 181.0$, $p < 0.001$ for analytic approach A and $Q(43) = 161.4$, $p < 0.001$ for analytic approach B).

For some of the analysis proposed in the RTs' pre-analysis plans, we realized ex post that the pre-registered specifications in analytic approach A cannot be implemented without ambiguity. For the sake of transparency, we report these cases and how we dealt with the uncertainty below. All of the listed issues have been double-checked with the respective RT.

- **`GFL12`**: The team specifies three exclusion criteria: participants should be excluded if (i) they have the same IP address, (ii) if they fail an attention check question, and (iii) if they spend less than 10 seconds on each instructions page. The dataset delivered by the team does neither include records of participants' IP addresses nor records on the time spent on each page of the experiment. Therefore, the only exclusion criterion that could be implemented for analytic approach A is (ii).
- **`JSV33`**: The pre-specified analysis of the team is not explicit about whether the control variable "education" should be treated to be ordinally scaled (six levels) or linearly scaled. We interpret the team's specification as if education should be controlled for using five binary indicators.
- **`LEU04`**: The team's pre-analysis plan is inconsistent with respect to the definition of the dependent variable and the statistical test of the hypothesis. In particular, the team defines the dependent variable as follows (page 3): *"We will calculate a continuous outcome variable for each participant based on the number of most-experienced candidates selected for older and female candidates (from 0 – 16)."* On page 4, they specify the following ordinary least squares regression model: *"y = minority candidate selected (1 = yes, 0 = no), x1 = competition treatment (1 = competition, 0 = control), x2 = minority characteristic (1 = woman, 0 = older male), clustered standard errors by participant."* We consulted with the team and agreed on relying on the definition of the dependent variable. Thus, for analytic approach A, we do not control for the minority characteristics (since it is not possible to do so with an aggregate score entering the model as dependent variable) and do not cluster standard errors on the participant level (as there is only one observation per participant).

» **NJP79**: Participants who timed-out on the decision page are excluded as the records on their offers are programmatically set to zero (these participants are excluded in all three analytic approaches A–C).

» **PEH91**: The team specifies exclusion criteria in the PAP subsection on the outcome variable (page 3), but not in the subsection dedicated to exclusion criteria. In particular, the design includes a set of three manipulation check questions and one control question; participants who fail to answer any of these questions correctly should be excluded according to the RT. We implemented these exclusion criteria (leading to 228 of 435 observations being excluded in analytic approaches A and C; note that these exclusion criteria do not affect analytic approach B). Three participants who did not respond to all questions used to construct the outcome measure (despite having submitted a valid Prolific completion code) are excluded in all analyses as we pre-registered that participants that fail to respond to the question/decision used to construct the outcome measure will be excluded (there is some ambiguity on this pre-registered exclusion criteria as it does not explicitly mention outcome measures based on multiple question where participants respond to some of these questions, but we interpreted this exclusion criteria as implying that participants needed to answer all the questions used to construct the outcome measure to be included).

» **SBL89**: In their pre-analysis plan, the team states: *"The intended donation [..] is the dependent variable and the [..] treatment [..] is the main explanatory variable (similar to a two sided t-test). We additionally include controls for socio-demographic variables (age, gender, household income) [..] as robustness checks."* As the primary analysis entering analytic approach A, we thus use the specification without controls. We also use robust standard errors even though the RT does not explicitly mention that they will use robust standard errors in the primary analysis, but they mention that they will use robust standard errors in the robustness checks with control variables (and after checking with the RT, they confirmed that they intended to use robust standard errors also in their primary analysis).

» **XKM55**: Five participants who do not complete all 25 trials used to construct the outcome measure (despite having submitted a valid Prolific completion code) are excluded in all analyses as we pre-registered that participants that fail to respond to the question/decision used to construct the outcome measure will be excluded (there is some ambiguity on this pre-registered exclusion criteria as it does not explicitly mention outcome measures based on multiple question where participants respond to some of these questions, but we interpreted this exclusion criteria as implying that participants needed to answer all the questions used to construct the outcome measure to be included).

» **ZKI49**: The team's proposed analysis involves the inclusion of perfectly collinear control variables. For each of the (original) control variables, one of the categories has been omitted (base category). Furthermore, the team registers that *"If some categories in gender, marital status, education and employment status have too few observations, they should be merged. Example, if out of 400 participants, only 10 specify nonbinary and 10 say prefer not to say, then x53 can be dropped."* We implement the analysis in analytic approach A as proposed by the team. In particular, the following categories have been merged: "non-binary" (n = 8) has been merged with "male" for the variable "gender"; "divorced/seperated" (n = 14) and "widowed" (n = 2) have been merged with "single" for the variable "marital status"; "less than high school" (n = 5) has been merged with "high school graduate" for the variable education; "doctorate degree" (n = 14) has been merged with "master degree" for the variable "eduation"; and "retired" (n = 14) has been merged with "unemployed" for the variable "employment". These mergers leave us (for analytic approach A) with one binary control variable for gender ("female"), two binary control variables for marital status ("in a relationship", "married"), three binary control variables for education ("bachelor degree", "technical/vocational training", "master/doctorate degree"), and three binary control variables for employment ("student", "employed part time", "employed full time").

» **ZZS69**: The team specified that standard errors should be clustered on the session level. As it is not clear how to define a "session" in the data collection, we consulted with the team and they indicated that the clustering was a residual of an initial version of their design and is obsolete for the final version of their experiment (and we therefore did not implement any clustering).

In the pre-registered exploratory analysis, where we estimate results separately for the top and bottom 50% quality designs (based on the average demeaned peer quality ratings), we did not pre-register how to divide all designs into two groups in case of an uneven number of designs (the analysis was pre-registered prior to knowing the exact number of designs/RTs in the data collection). As we collected data for 45 designs, the top and bottom 50% groups will not contain an identical number of designs. We decided to include 22 designs in the top group and 23 designs in the bottom group as we had pre-registered that in case of ties in the quality rating at the median rating all tied designs would be included in the bottom 50% group. As this was a similar decision, we decided to follow the same principle. Yet, this decision was not important for our results. The effect size of the median design (QLM89), in terms of the peer assessment, was 0.051 (95% CI [−0.197, 0.299], $p = 0.687$) in analytic approach A and 0.074 (95% CI [−0.210, 0.360], $p = 0.605$) for analytic approach B. If this design is placed in the top 50% quality group instead, the meta-analytic effect size is −0.047 (95% CI [−0.109, 0.015], $p = 0.137$) for analytic approach A and −0.043 (95% CI [−0.104, 0.017], $p = 0.159$) for analytic approach B in the 50% top rated designs; and the meta-analytic effect size is −0.128 (95% CI [−0.235, −0.022], $p = 0.018$) for analytic approach A and −0.132 (95% CI [−0.228, −0.035], $p = 0.008$) for analytic approach B in the 50% bottom rated designs. The estimated τ is 0.104 (95% CI [0.042, 0.187]) for analytic approach A and 0.098 (95% CI [0.035, 0.184]) for analytic approach B in the 50% top rated designs, and 0.238 (95% CI [0.177, 0.366]) for analytic approach A and 0.212 (95% CI [0.169, 0.358]) for analytic approach B in the 50% bottom rated designs. And the result of the heterogeneity test is $Q(21) = 41.6$ ($p = 0.005$) for analytic approach A and $Q(21) = 39.4$ ($p = 0.009$) for analytic approach B in the 50% top rated designs, and $Q(22) = 135.6$ ($p < 0.001$) for analytic approach A and $Q(22) = 117.0$ ($p < 0.001$) for analytic approach B in the 50% bottom rated designs.

SI Tables

Table S1. Individual study results based on analytic approach A. Reported are the effect size estimate (in Cohen's *d* units), the standard error, the *p*-value, the number of observations (after any exclusions), the 95% CI, as well as the average peer quality assessment (before and after demeaning the scores per RT) for each of the 45 research designs (sorted alphabetically by the research teams' ID). * $p < 0.05$, ** $p < 0.005$.

Team ID	Cohen's *d*	Standard Error	*p*-Value	No. of Obs.†	95% CI	Avg. Peer Rating	Rating Demeaned
ACH91	0.129	0.101	0.203	394	[-0.070, 0.328]	6.10	0.29
BDQ29	0.011	0.134	0.937	236	[-0.253, 0.274]	6.40	0.17
BXE54	-0.132	0.099	0.185	406	[-0.327, 0.063]	5.90	-0.25
GFL12	-0.123	0.101	0.225	393	[-0.321, 0.076]	7.30	0.93
GWP43	-0.315	0.105	0.003**	1053	[-0.522,-0.108]	4.30	-1.66
HCA40	0.158	0.097	0.102	425	[-0.032, 0.348]	7.30	1.50
HTZ90	-0.006	0.098	0.952	415	[-0.199, 0.187]	7.20	0.91
HYA54	-0.143	0.102	0.164	383	[-0.344, 0.059]	6.80	0.69
ICP06	0.069	0.101	0.492	393	[-0.129, 0.268]	5.70	-0.54
IZU58	-0.159	0.107	0.139	345	[-0.370, 0.052]	5.00	-1.03
JSV33	-0.023	0.094	0.808	413	[-0.208, 0.162]	7.20	1.04
JTI38	0.012	0.098	0.900	418	[-0.180, 0.205]	5.90	0.08
JUZ91	-0.158	0.100	0.114	413	[-0.354, 0.038]	5.50	-0.19
KLX01	-0.157	0.099	0.113	410	[-0.351, 0.037]	6.50	0.38
LCA31	-0.111	0.099	0.262	410	[-0.306, 0.084]	7.20	0.94
LEU04	-0.141	0.099	0.156	407	[-0.336, 0.054]	4.50	-1.75
LGT85	-0.095	0.100	0.344	397	[-0.292, 0.102]	5.20	-0.40
MUE79	-0.142	0.097	0.147	406	[-0.333, 0.050]	5.80	0.35
NCW80	0.150	0.113	0.184	319	[-0.072, 0.373]	6.60	0.54
NJJ10	-0.177	0.098	0.071	427	[-0.370, 0.015]	6.40	0.13
NJP79	-0.056	0.108	0.604	268	[-0.269, 0.157]	7.00	1.37
OUU63	-0.056	0.113	0.621	312	[-0.280, 0.167]	7.10	0.78
PCS27	-0.023	0.097	0.812	428	[-0.213, 0.167]	4.10	-1.56
PEH91	-0.035	0.139	0.804	207	[-0.308, 0.239]	5.80	-0.48
PKY70	-0.185	0.109	0.091	376	[-0.399, 0.030]	6.10	-0.10
PRF65	-0.199	0.097	0.041*	409	[-0.391,-0.008]	5.20	-1.22

continued on next page

Team ID	Cohen's *d*	Standard Error	*p*-Value	No. of Obs.†	95% CI	Avg. Peer Rating	Rating Demeaned
QLM89	0.051	0.126	0.687	191	[-0.197, 0.299]	5.60	0.16
RDP12	0.459	0.100	0.000**	397	[0.262, 0.657]	5.20	-1.12
RPZ39	0.032	0.099	0.749	396	[-0.163, 0.227]	6.00	0.10
RZU75	-0.516	0.177	0.004**	150	[-0.866,-0.165]	4.80	-1.46
SBL89	-0.210	0.100	0.035*	404	[-0.406,-0.015]	5.80	-0.04
TEQ73	-0.142	0.098	0.149	413	[-0.336, 0.051]	6.40	-0.11
TVX41	-0.377	0.087	0.000**	417	[-0.549,-0.205]	6.60	0.53
VHJ19	-0.005	0.100	0.961	400	[-0.202, 0.192]	3.40	-2.45
WDO94	0.015	0.098	0.877	400	[-0.178, 0.209]	6.70	0.53
XAI09	0.105	0.103	0.307	381	[-0.097, 0.307]	4.20	-1.21
XKM55	-1.048	0.109	0.000**	350	[-1.262,-0.835]	5.20	-0.68
XKZ34	-0.055	0.100	0.582	404	[-0.251, 0.141]	7.20	1.21
XUW82	-0.241	0.108	0.027*	339	[-0.455,-0.028]	5.10	-1.31
XZK69	-0.044	0.112	0.695	317	[-0.265, 0.177]	5.80	0.26
XZZ66	0.356	0.180	0.050*	130	[0.000, 0.712]	6.70	0.66
YPZ45	-0.020	0.104	0.849	371	[-0.224, 0.184]	7.80	1.48
ZKI49	-0.068	0.101	0.503	407	[-0.266, 0.131]	6.40	0.42
ZZS69	0.141	0.141	0.319	199	[-0.137, 0.420]	5.90	0.33
ZZW48	-0.271	0.099	0.006*	410	[-0.465,-0.077]	7.40	1.77

† Number of observations used in the ordinary least squares regressions. All but one team use one observation per participant. The design by GWP43 involves three observations per subject (with standard errors being clustered at the participant level), i.e., 351 (participants) × 3 (items) = 1,053 observations.

Table S2. Individual study results based on analytic approach B. Reported are the effect size estimate (in Cohen's *d* units), the standard error, the *p*-value, the number of observations (after any exclusions), the 95% CI, as well as the average peer quality assessment (before and after demeaning the scores per RT) for each of the 45 research designs (sorted alphabetically by the research teams' ID). * $p < 0.05$, ** $p < 0.005$.

Team ID	Cohen's *d*	Standard Error	*p*-Value	No. of Obs.	95% CI	Avg. Peer Rating	Rating Demeaned
ACH91	0.146	0.101	0.150	395	[-0.053, 0.345]	6.10	0.29
BDQ29	0.008	0.138	0.954	237	[-0.264, 0.280]	6.40	0.17
BXE54	-0.132	0.099	0.185	406	[-0.327, 0.063]	5.90	-0.25
GFL12	-0.100	0.097	0.302	428	[-0.290, 0.090]	7.30	0.93
GWP43	-0.328	0.098	0.001**	421	[-0.521,-0.136]	4.30	-1.66
HCA40	0.153	0.097	0.115	426	[-0.037, 0.343]	7.30	1.50
HTZ90	-0.017	0.097	0.864	423	[-0.207, 0.174]	7.20	0.91
HYA54	-0.137	0.097	0.160	427	[-0.327, 0.054]	6.80	0.69
ICP06	0.035	0.099	0.720	412	[-0.158, 0.229]	5.70	-0.54
IZU58	-0.061	0.100	0.545	401	[-0.257, 0.136]	5.00	-1.03
JSV33	-0.041	0.098	0.679	414	[-0.234, 0.153]	7.20	1.04
JTI38	0.012	0.098	0.900	418	[-0.180, 0.205]	5.90	0.08
JUZ91	-0.214	0.099	0.030*	413	[-0.408,-0.021]	5.50	-0.19
KLX01	-0.157	0.099	0.113	410	[-0.351, 0.037]	6.50	0.38
LCA31	-0.114	0.099	0.249	410	[-0.308, 0.080]	7.20	0.94
LEU04	-0.141	0.099	0.157	407	[-0.336, 0.054]	4.50	-1.75
LGT85	-0.107	0.097	0.271	428	[-0.297, 0.084]	5.20	-0.40
MUE79	-0.161	0.099	0.105	406	[-0.355, 0.034]	5.80	0.35
NCW80	0.131	0.098	0.182	419	[-0.061, 0.324]	6.60	0.54
NJJ10	-0.188	0.097	0.053	427	[-0.378, 0.002]	6.40	0.13
NJP79	-0.032	0.122	0.793	268	[-0.273, 0.209]	7.00	1.37
OUU63	-0.024	0.113	0.831	312	[-0.247, 0.199]	7.10	0.78
PCS27	-0.023	0.097	0.812	428	[-0.213, 0.167]	4.10	-1.56
PEH91	-0.175	0.095	0.067	432	[-0.362, 0.013]	5.80	-0.48
PKY70	-0.192	0.103	0.063	376	[-0.395, 0.010]	6.10	-0.10
PRF65	-0.181	0.099	0.068	411	[-0.375, 0.013]	5.20	-1.22
QLM89	0.075	0.144	0.605	192	[-0.210, 0.360]	5.60	0.16
RDP12	0.459	0.100	0.000**	397	[0.262, 0.656]	5.20	-1.12
RPZ39	0.026	0.100	0.794	396	[-0.171, 0.223]	6.00	0.10

continued on next page

Team ID	Cohen's *d*	Standard Error	*p*-Value	No. of Obs.	95% CI	Avg. Peer Rating	Rating Demeaned
RZU75	-0.472	0.160	0.004**	150	[-0.789,-0.155]	4.80	-1.46
SBL89	-0.171	0.097	0.078	425	[-0.362, 0.019]	5.80	-0.04
TEQ73	-0.142	0.098	0.149	413	[-0.336, 0.051]	6.40	-0.11
TVX41	-0.379	0.098	0.000**	417	[-0.572,-0.187]	6.60	0.53
VHJ19	-0.005	0.100	0.961	400	[-0.202, 0.192]	3.40	-2.45
WDO94	0.014	0.100	0.886	400	[-0.183, 0.211]	6.70	0.53
XAI09	0.066	0.100	0.513	398	[-0.132, 0.263]	4.20	-1.21
XKM55	-1.048	0.125	0.000**	350	[-1.294,-0.802]	5.20	-0.68
XKZ34	-0.059	0.099	0.556	405	[-0.254, 0.137]	7.20	1.21
XUW82	-0.249	0.099	0.013*	407	[-0.444,-0.054]	5.10	-1.31
XZK69	-0.048	0.103	0.639	376	[-0.251, 0.154]	5.80	0.26
XZZ66	0.356	0.180	0.050*	130	[0.000, 0.712]	6.70	0.66
YPZ45	-0.004	0.100	0.969	396	[-0.200, 0.192]	7.80	1.48
ZKI49	-0.041	0.099	0.679	407	[-0.236, 0.154]	6.40	0.42
ZZS69	0.122	0.142	0.390	199	[-0.157, 0.401]	5.90	0.33
ZZW48	-0.271	0.099	0.006*	410	[-0.465,-0.077]	7.40	1.77

Table S3. Individual study results based on analytic approach C. Reported are the effect size estimate (in Cohen's *d* units), the standard error, the *p*-value, the number of observations (after any exclusions), the 95% CI, as well as the average peer quality assessment (before and after demeaning the scores per RT) for each of the 45 research designs (sorted alphabetically by the research teams' ID). * $p < 0.05$, ** $p < 0.005$.

Team ID	Cohen's *d*	Standard Error	*p*-Value	No. of Obs.	95% CI	Avg. Peer Rating	Rating Demeaned
ACH91	0.129	0.101	0.203	394	[-0.070, 0.328]	6.10	0.29
BDQ29	0.008	0.138	0.954	237	[-0.264, 0.280]	6.40	0.17
BXE54	-0.132	0.099	0.185	406	[-0.327, 0.063]	5.90	-0.25
GFL12	-0.123	0.101	0.225	393	[-0.321, 0.076]	7.30	0.93
GWP43	-0.315	0.105	0.003**	351	[-0.522,-0.108]	4.30	-1.66
HCA40	0.153	0.097	0.115	426	[-0.037, 0.343]	7.30	1.50
HTZ90	-0.006	0.098	0.952	415	[-0.198, 0.186]	7.20	0.91
HYA54	-0.143	0.102	0.164	383	[-0.344, 0.059]	6.80	0.69
ICP06	0.069	0.101	0.492	393	[-0.129, 0.268]	5.70	-0.54
IZU58	-0.141	0.108	0.191	345	[-0.354, 0.071]	5.00	-1.03
JSV33	-0.041	0.098	0.679	414	[-0.234, 0.153]	7.20	1.04
JTI38	0.012	0.098	0.900	418	[-0.180, 0.205]	5.90	0.08
JUZ91	-0.214	0.099	0.030*	413	[-0.408,-0.021]	5.50	-0.19
KLX01	-0.157	0.099	0.113	410	[-0.351, 0.037]	6.50	0.38
LCA31	-0.114	0.099	0.249	410	[-0.308, 0.080]	7.20	0.94
LEU04	-0.141	0.099	0.157	407	[-0.336, 0.054]	4.50	-1.75
LGT85	-0.095	0.101	0.345	397	[-0.293, 0.103]	5.20	-0.40
MUE79	-0.161	0.099	0.105	406	[-0.355, 0.034]	5.80	0.35
NCW80	0.112	0.111	0.313	325	[-0.106, 0.330]	6.60	0.54
NJJ10	-0.188	0.097	0.053	427	[-0.378, 0.002]	6.40	0.13
NJP79	-0.032	0.122	0.793	268	[-0.273, 0.209]	7.00	1.37
OUU63	-0.024	0.113	0.831	312	[-0.247, 0.199]	7.10	0.78
PCS27	-0.023	0.097	0.812	428	[-0.213, 0.167]	4.10	-1.56
PEH91	-0.035	0.138	0.802	207	[-0.307, 0.238]	5.80	-0.48
PKY70	-0.192	0.103	0.063	376	[-0.395, 0.010]	6.10	-0.10
PRF65	-0.181	0.099	0.068	411	[-0.375, 0.013]	5.20	-1.22
QLM89	0.075	0.144	0.605	192	[-0.210, 0.360]	5.60	0.16
RDP12	0.459	0.100	0.000**	397	[0.262, 0.656]	5.20	-1.12
RPZ39	0.026	0.100	0.794	396	[-0.171, 0.223]	6.00	0.10

continued on next page

Team ID	Cohen's *d*	Standard Error	*p*-Value	No. of Obs.	95% CI	Avg. Peer Rating	Rating Demeaned
RZU75	-0.472	0.160	0.004**	150	[-0.789,-0.155]	4.80	-1.46
SBL89	-0.210	0.100	0.035*	404	[-0.406,-0.015]	5.80	-0.04
TEQ73	-0.142	0.098	0.149	413	[-0.336, 0.051]	6.40	-0.11
TVX41	-0.379	0.098	0.000**	417	[-0.572,-0.187]	6.60	0.53
VHJ19	-0.005	0.100	0.961	400	[-0.202, 0.192]	3.40	-2.45
WDO94	0.014	0.100	0.886	400	[-0.183, 0.211]	6.70	0.53
XAI09	0.105	0.103	0.307	381	[-0.097, 0.307]	4.20	-1.21
XKM55	-1.048	0.125	0.000**	350	[-1.294,-0.802]	5.20	-0.68
XKZ34	-0.055	0.100	0.582	404	[-0.251, 0.141]	7.20	1.21
XUW82	-0.241	0.108	0.027*	339	[-0.455,-0.028]	5.10	-1.31
XZK69	-0.044	0.113	0.696	317	[-0.265, 0.177]	5.80	0.26
XZZ66	0.356	0.180	0.050*	130	[0.000, 0.712]	6.70	0.66
YPZ45	-0.020	0.102	0.847	371	[-0.221, 0.181]	7.80	1.48
ZKI49	-0.041	0.099	0.679	407	[-0.236, 0.154]	6.40	0.42
ZZS69	0.122	0.142	0.390	199	[-0.157, 0.401]	5.90	0.33
ZZW48	-0.271	0.099	0.006*	410	[-0.465,-0.077]	7.40	1.77

Table S4. Individual study results based on analytic approach B with clustering on the batch level. Reported are the effect size estimate (in Cohen's *d* units), the standard error, the *p*-value, the number of observations (after any exclusions), the 95% CI, as well as the average peer quality assessment (before and after demeaning the scores per RT) for each of the 45 research designs (sorted alphabetically by the research teams' ID). * $p < 0.05$, ** $p < 0.005$.

Team ID	Cohen's *d*	Standard Error	*p*-Value	No. of Obs.	95% CI	Avg. Peer Rating	Rating Demeaned
ACH91	0.146	0.103	0.158	395	[-0.058, 0.349]	6.10	0.29
BDQ29	0.008	0.134	0.952	237	[-0.259, 0.275]	6.40	0.17
BXE54	-0.132	0.100	0.189	406	[-0.330, 0.066]	5.90	-0.25
GFL12	-0.100	0.094	0.288	428	[-0.285, 0.085]	7.30	0.93
GWP43	-0.328	0.098	0.001**	421	[-0.521,-0.135]	4.30	-1.66
HCA40	0.153	0.098	0.120	426	[-0.040, 0.346]	7.30	1.50
HTZ90	-0.017	0.105	0.874	423	[-0.224, 0.191]	7.20	0.91
HYA54	-0.137	0.096	0.156	427	[-0.326, 0.053]	6.80	0.69
ICP06	0.035	0.104	0.733	412	[-0.170, 0.241]	5.70	-0.54
IZU58	-0.061	0.102	0.554	401	[-0.263, 0.142]	5.00	-1.03
JSV33	-0.041	0.095	0.670	414	[-0.229, 0.148]	7.20	1.04
JTI38	0.012	0.100	0.901	418	[-0.185, 0.210]	5.90	0.08
JUZ91	-0.214	0.102	0.038*	413	[-0.416,-0.012]	5.50	-0.19
KLX01	-0.157	0.102	0.126	410	[-0.359, 0.045]	6.50	0.38
LCA31	-0.114	0.099	0.252	410	[-0.310, 0.082]	7.20	0.94
LEU04	-0.141	0.104	0.178	407	[-0.347, 0.065]	4.50	-1.75
LGT85	-0.107	0.110	0.334	428	[-0.324, 0.111]	5.20	-0.40
MUE79	-0.161	0.106	0.134	406	[-0.372, 0.050]	5.80	0.35
NCW80	0.131	0.094	0.167	419	[-0.056, 0.318]	6.60	0.54
NJJ10	-0.188	0.094	0.047*	427	[-0.373,-0.003]	6.40	0.13
NJP79	-0.032	0.121	0.792	268	[-0.273, 0.209]	7.00	1.37
OUU63	-0.024	0.113	0.831	312	[-0.249, 0.200]	7.10	0.78
PCS27	-0.023	0.097	0.813	428	[-0.216, 0.170]	4.10	-1.56
PEH91	-0.175	0.090	0.055	432	[-0.353, 0.004]	5.80	-0.48
PKY70	-0.192	0.108	0.077	376	[-0.406, 0.021]	6.10	-0.10
PRF65	-0.181	0.100	0.074	411	[-0.379, 0.018]	5.20	-1.22
QLM89	0.075	0.148	0.614	192	[-0.218, 0.368]	5.60	0.16
RDP12	0.459	0.096	0.000**	397	[0.270, 0.648]	5.20	-1.12
RPZ39	0.026	0.104	0.802	396	[-0.180, 0.232]	6.00	0.10

continued on next page

Team ID	Cohen's *d*	Standard Error	*p*-Value	No. of Obs.	95% CI	Avg. Peer Rating	Rating Demeaned
RZU75	-0.472	0.154	0.003**	150	[-0.777,-0.167]	4.80	-1.46
SBL89	-0.171	0.085	0.046*	425	[-0.339,-0.003]	5.80	-0.04
TEQ73	-0.142	0.089	0.112	413	[-0.318, 0.034]	6.40	-0.11
TVX41	-0.379	0.099	0.000**	417	[-0.575,-0.184]	6.60	0.53
VHJ19	-0.005	0.100	0.961	400	[-0.203, 0.193]	3.40	-2.45
WDO94	0.014	0.096	0.881	400	[-0.175, 0.204]	6.70	0.53
XAI09	0.066	0.103	0.526	398	[-0.139, 0.270]	4.20	-1.21
XKM55	-1.048	0.175	0.000**	350	[-1.395,-0.702]	5.20	-0.68
XKZ34	-0.059	0.108	0.589	405	[-0.273, 0.156]	7.20	1.21
XUW82	-0.249	0.095	0.010*	407	[-0.437,-0.060]	5.10	-1.31
XZK69	-0.048	0.098	0.621	376	[-0.242, 0.145]	5.80	0.26
XZZ66	0.356	0.163	0.032*	130	[0.032, 0.681]	6.70	0.66
YPZ45	-0.004	0.099	0.969	396	[-0.199, 0.191]	7.80	1.48
ZKI49	-0.041	0.099	0.678	407	[-0.236, 0.154]	6.40	0.42
ZZS69	0.122	0.137	0.374	199	[-0.149, 0.393]	5.90	0.33
ZZW48	-0.271	0.104	0.010*	410	[-0.477,-0.065]	7.40	1.77

Table S5. Choice points per research team. Reported are the conceptualization of the dependent variable (moral behavior) and operationalization of the treatment intervention (competition) for each of the 45 research designs. Moral behavior is categorized into four groups: *(i)* donations to charity; *(ii)* generosity to other player(s) in the experiment; *(iii)* cheating/deception; and *(iv)* “other designs,” i.e., designs that cannot be classified into the other three groups. The operationalization of the competition intervention has been delineated along two binary variables: the first competition variable captures whether the competition involves monetary incentives; the second variable captures whether or not the competition is directly linked to moral behavior, i.e., whether moral behavior as conceptualized by the teams affects the likelihood of winning (or the rank in) the competition.

	Moral Behavior				Competition	
**Team ID**	**Charity**	**Generosity**	**Cheating**	**Other**	**Monetary**	**Linked**
ACH91	✗	✗	✓	✗	✓	✓
BDQ29	✗	✓	✗	✗	✗	✗
BXE54	✗	✗	✓	✗	✗	✗
GFL12	✗	✗	✓	✗	✓	✗
GWP43	✗	✗	✗	✓	✗	✗
HCA40	✓	✗	✗	✗	✓	✗
HTZ90	✗	✗	✓	✗	✓	✓
HYA54	✗	✗	✓	✗	✓	✓
ICP06	✓	✗	✗	✗	✓	✗
IZU58	✓	✗	✗	✗	✓	✗
JSV33	✓	✗	✗	✗	✓	✓
JTI38	✗	✗	✓	✗	✓	✓
JUZ91	✗	✗	✓	✗	✓	✓
KLX01	✗	✗	✓	✗	✓	✓
LCA31	✗	✗	✓	✗	✓	✓
LEU04	✗	✗	✗	✓	✓	✓
LGT85	✗	✓	✗	✗	✗	✓
MUE79	✓	✗	✗	✗	✓	✓
NCW80	✗	✓	✗	✗	✓	✗
NJJ10	✗	✗	✓	✗	✓	✓
NJP79	✗	✗	✓	✗	✓	✓
OUU63	✗	✗	✓	✗	✓	✗
PCS27	✗	✗	✓	✗	✗	✓
PEH91	✗	✗	✗	✓	✓	✗

continued on next page

	Moral Behavior			Competition		
**Team ID**	**Charity**	**Generosity**	**Cheating**	**Other**	**Monetary**	**Linked**
PKY70	×	✓	×	×	×	×
PRF65	×	×	✓	×	✓	✓
QLM89	×	✓	×	×	✓	×
RDP12	✓	×	×	×	✓	✓
RPZ39	×	×	✓	×	✓	✓
RZU75	×	×	×	✓	×	×
SBL89	×	×	✓	×	✓	✓
TEQ73	×	×	✓	×	✓	×
TVX41	×	×	✓	×	✓	✓
VHJ19	✓	×	×	×	✓	✓
WDO94	×	×	✓	×	✓	✓
XAI09	×	✓	×	×	×	×
XKM55	×	×	✓	×	✓	✓
XKZ34	✓	×	×	×	✓	×
XUW82	✓	×	×	×	×	×
XZK69	×	×	✓	×	✓	✓
XZZ66	×	✓	×	×	✓	✓
YPZ45	×	×	✓	×	✓	✓
ZKI49	×	×	✓	×	✓	×
ZZS69	×	✓	×	×	×	×
ZZW48	×	✓	×	×	✓	✓

Table S6. Meta-regressions on common design choices for analytic approach A and B. Reported are the results of meta-regressions testing for moderating effects of research teams' conceptualization of moral behavior and research teams' operationalization of the competition intervention for analytic approaches A and B, respectively. The table reports coefficient estimates and standard errors (in parentheses) alongside the corresponding *z*- and *p*-values (in brackets). Intervals reported for the residual τ and the residual I^2 correspond to 95% CIs. * $p < 0.05$, ** $p < 0.05$.

	**Analytic Approach A**		**Analytic Approach B**	
	***b* (*se*)**	***z* [*p*]**	***b* (*se*)**	***z* [*p*]**
Donation to charity	0.213<br>(0.135)	1.577<br>[0.115]	0.241*****<br>(0.121)	1.989<br>[0.047]
Generosity to other player(s)	0.259<br>(0.134)	1.942<br>[0.052]	0.277*****<br>(0.120)	2.297<br>[0.022]
Cheating / deception	0.076<br>(0.127)	0.596<br>[0.551]	0.114<br>(0.114)	0.999<br>[0.318]
Monetary competition	0.095<br>(0.092)	1.034<br>[0.301]	0.099<br>(0.084)	1.172<br>[0.241]
Moral behavior → competition	–0.017<br>(0.074)	–0.229<br>[0.819]	–0.022<br>(0.068)	–0.325<br>[0.745]
Constant	–0.282*****<br>(0.121)	–2.332<br>[0.020]	–0.310******<br>(0.108)	–2.865<br>[0.004]
Residual τ	0.182<br>[0.158, 0.284]		0.163<br>[0.152, 0.279]	
Residual I^2	75.1%<br>[69.6, 88.1]		71.3%<br>[68.4, 87.9]	
Residual *Q*	$Q(39) = 156.9$<br>$p < 0.001$		$Q(39) = 136.0$<br>$p < 0.001$	
R^2	3.109%		6.420%	
Number of observations	45		45	

Notes: Moral behavior is categorized into four groups: *(i)* donations to charity; *(ii)* generosity to other player(s); *(iii)* cheating/deception; and *(iv)* other designs, which constitutes the baseline category in the meta-regression. The operationalization of the competition intervention is delineated along two binary variables: *(i)* whether the competition involves monetary incentives; and *(ii)* whether the competition is directly linked to moral behavior, i.e., whether moral behavior affects the likelihood of winning (or the rank in) the competition.

Table S7. Attrition rates separated by treatment conditions. Reported are attrition rates (in %), defined as participants who provided informed consent, completed the common attention check, and were successfully redirected to one of the 45 designs but failed to complete the entire experiment (i.e., did not provide a valid Prolific completion code). The two rightmost columns report the test statistic (*z*-value) and the corresponding *p*-value of probit regressions of attrition on a treatment indicator, clustering standard errors on the batch level (i.e., the level of randomization) to account for correlations in dropouts in designs using simultaneous group interactions. * $p < 0.05$, ** $p < 0.05$.

	**Attrition Rates in %**		**Comparison of Conditions**	
**Team ID**	**Control Condition**	**Competition Condition**	***z*-Value**	***p*-Value**
ACH91	10.70	9.38	0.439	0.661
BDQ29	58.26	35.81	3.055**	0.002
BXE54	5.58	5.14	0.166	0.868
GFL12	6.22	4.41	0.721	0.471
GWP43	2.70	6.82	1.761	0.078
HCA40	4.09	4.42	0.146	0.884
HTZ90	7.56	5.29	0.853	0.394
HYA54	5.26	5.80	0.194	0.846
ICP06	6.05	3.23	0.992	0.321
IZU58	9.05	7.41	0.525	0.599
JSV33	4.61	3.72	0.398	0.691
JTI38	3.70	4.11	0.218	0.828
JUZ91	5.36	3.37	0.888	0.375
KLX01	3.74	5.56	0.716	0.474
LCA31	5.48	4.63	0.345	0.730
LEU04	3.37	5.50	1.052	0.293
LGT85	7.49	3.54	1.733	0.083
MUE79	7.34	4.02	1.437	0.151
NCW80	8.22	6.03	0.714	0.475
NJJ10	4.50	4.02	0.205	0.838
NJP79	18.86	16.82	0.412	0.680
OUU63	15.28	11.21	0.940	0.347
PCS27	3.59	3.62	0.015	0.988
PEH91	5.02	5.02	0.001	0.999
PKY70	4.06	6.50	0.874	0.382

continued on next page

Team ID	Attrition Rates in %		Comparison of Conditions	
	Control Condition	Competition Condition	*z*-Value	*p*-Value
PRF65	5.58	5.45	0.044	0.965
QLM89	6.19	3.41	0.997	0.319
RDP12	8.96	8.11	0.269	0.788
RPZ39	10.05	10.34	0.095	0.925
RZU75	3.85	5.58	0.753	0.451
SBL89	4.00	6.70	1.115	0.265
TEQ73	7.21	7.17	0.010	0.992
TVX41	4.15	10.64	2.466*	0.014
VHJ19	6.54	5.66	0.275	0.783
WDO94	5.63	6.13	0.159	0.873
XAI09	12.00	6.39	1.820	0.069
XKM55	5.91	26.73	3.894**	0.000
XKZ34	8.41	6.70	0.522	0.602
XUW82	4.63	6.51	0.707	0.480
XZK69	12.56	13.76	0.315	0.752
XZZ66	42.18	28.70	2.083*	0.037
YPZ45	6.48	8.49	0.618	0.536
ZKI49	7.69	5.29	0.866	0.386
ZZS69	4.19	5.31	0.434	0.664
ZZW48	6.64	3.62	1.192	0.233

S8. Meta-regression on an indicator for designs with differences in attrition rates between treatment conditions for analytic approach A and B. Reported are the results of meta-regressions testing whether designs involving (suggestive or significant; $p < 0.05$) differences in attrition rates differ systematically from designs not involving systematic differences in attrition, and whether (part of) the heterogeneity is attributable to attrition effects. The table reports coefficient estimates and standard errors (in parentheses) alongside the corresponding *z*- and *p*-values (in brackets). Intervals reported for the residual τ and the residual I^2 correspond to 95% CIs. * $p < 0.05$, ** $p < 0.05$.

	**Analytic Approach A**		**Analytic Approach B**	
	***b* (*se*)**	***z* [*p*]**	***b* (*se*)**	***z* [*p*]**
Difference in attrition rates (BDQ29, TVX41, XKM55, XZZ66)	–0.276***** (0.106)	–2.593 [0.010]	–0.260***** (0.106)	–2.463 [0.014]
Constant	–0.063***** (0.030)	–2.087 [0.037]	–0.066***** (0.029)	–2.292 [0.022]
Residual τ	0.162 [0.149, 0.264]		0.153 [0.142, 0.259]	
Residual I^2	70.4% [66.9, 86.4]		68.9% [65.5, 86.3]	
Residual *Q*	$Q(43) = 145.4$ $p < 0.001$		$Q(43) = 138.4$ $p < 0.001$	
R^2	23.555%		17.248%	
Number of observations	45		45	

SI Figures

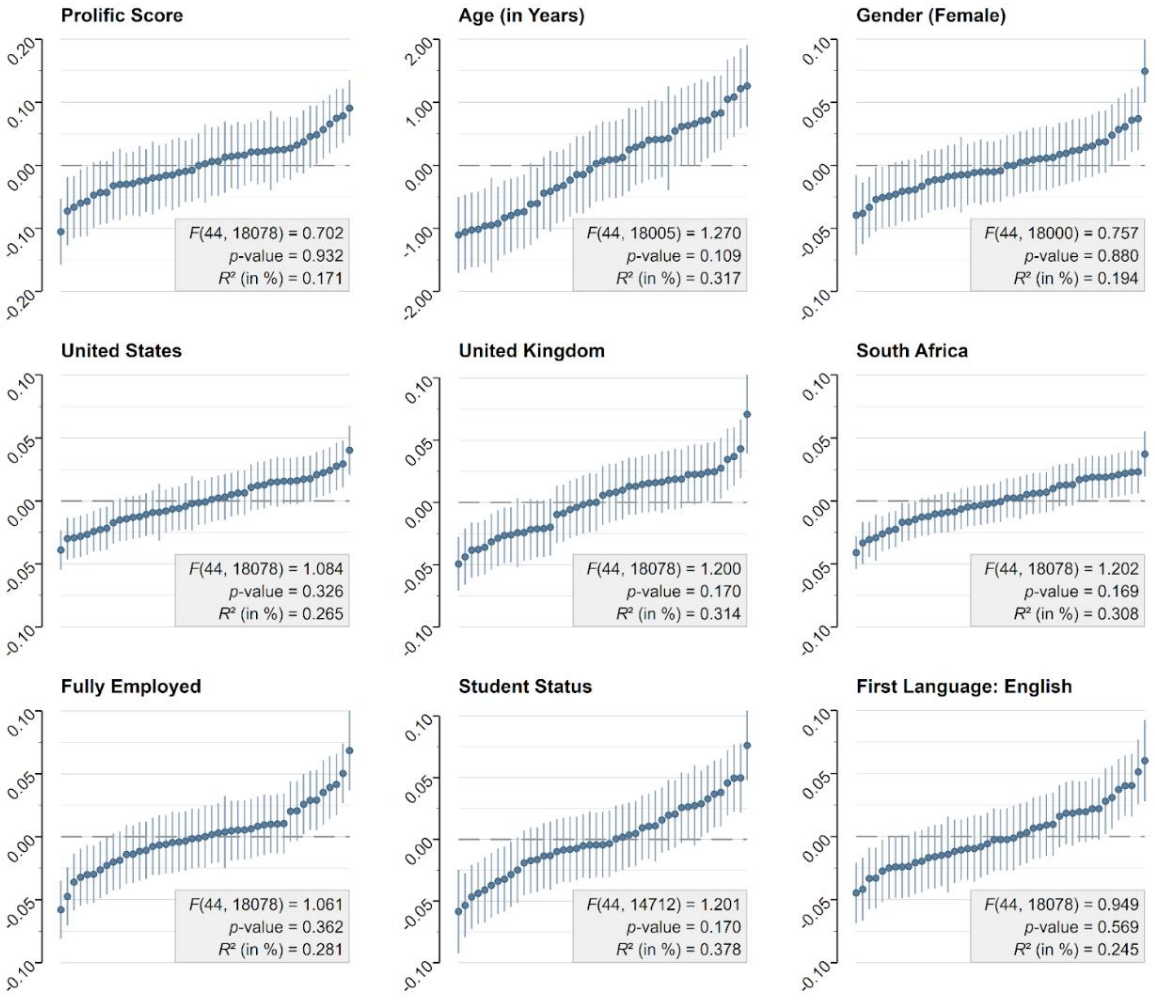

S1. Participant characteristics across the 45 research designs. Plotted are the means (and 95% CIs) of the de-meaned variables for participants that completed the experiment, i.e., the average deviation from the overall mean, for each of the 45 research designs. The *F*-statistics and the corresponding *p*-values, reported in the boxes within each panel, pertain to joint tests of fixed-effects in linear regressions; R^2 (in %) captures how much of the variance is explained by team fixed-effects.

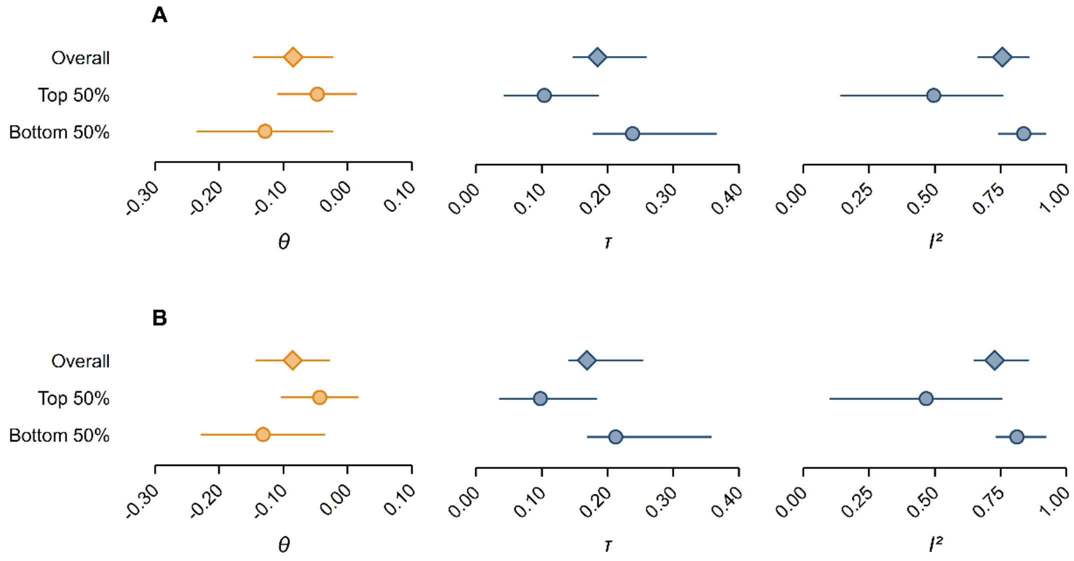

S2. Meta-analytic results for top and bottom 50% quality designs. (A) Plotted are 95% CIs of the meta-analytic effect size (left column) and the heterogeneity measures τ (middle column) and I^2 (right column) in the 50% top and bottom rated experimental designs and the overall sample for analytic approach A. The meta-analytic effect size is −0.047 in the top-rated designs and –0.128 in the bottom rated designs, the standard deviation of the true effect size across experimental designs (τ) is 0.104 in the top-rated designs and 0.238 in the bottom rated designs, and I^2 is 49.6% in the top-rated designs and 83.8% in the bottom rated designs. See Supporting Information, section 3 for more details. **(B)** Plotted are 95% CIs of the meta-analytic effect size (left column) and the heterogeneity measures τ (middle column) and I^2 (right column) in the 50% top and bottom rated experimental designs and the overall sample for analytic approach B. The meta-analytic effect size is −0.043 in the top-rated designs and –0.132 in the bottom rated designs, the standard deviation of the true effect size across experimental designs (τ) is 0.098 in the top-rated designs and 0.212 in the bottom rated designs, and I^2 is 49.7% in the top-rated designs and 81.2% in the bottom rated designs. See Supporting Information, section 3for more details.
